# Biodiversity intervention enhances immune regulation and health-associated commensal microbiota among daycare children

**DOI:** 10.1126/sciadv.aba2578

**Published:** 2020-10-14

**Authors:** Marja I. Roslund, Riikka Puhakka, Mira Grönroos, Noora Nurminen, Sami Oikarinen, Ahmad M. Gazali, Ondřej Cinek, Lenka Kramná, Nathan Siter, Heli K. Vari, Laura Soininen, Anirudra Parajuli, Juho Rajaniemi, Tuure Kinnunen, Olli H. Laitinen, Heikki Hyöty, Aki Sinkkonen, ADELE research group

**Affiliations:** 1 Ecosystems and Environment Research Programme, Faculty of Biological and Environmental Sciences, University of Helsinki, Niemenkatu 73, FI-15140 Lahti, Finland.; 2 Faculty of Medicine and Health Technology, Tampere University, Arvo Ylpön katu 34, FI-33520 Tampere, Finland.; 3 Department of Clinical Microbiology, Institute of Clinical Medicine, University of Eastern Finland, Kuopio, Finland.; 4 Department of Pediatrics, 2nd Faculty of Medicine, Charles University and University Hospital Motol, V Úvalu 84, Praha 5, 150 06 Prague, Czech Republic.; 5 Faculty of Built Environment, Tampere University, Korkeakoulunkatu 5, FI-33720 Tampere, Finland.; 6 Eastern Finland Laboratory Centre (ISLAB), Kuopio, Finland.; 7 Natural Resources Institute Finland Luke, Itäinen Pitkäkatu 4A, 20520 Turku, Finland.

## Abstract

As the incidence of immune-mediated diseases has increased rapidly in developed societies, there is an unmet need for novel prophylactic practices to fight against these maladies. This study is the first human intervention trial in which urban environmental biodiversity was manipulated to examine its effects on the commensal microbiome and immunoregulation in children. We analyzed changes in the skin and gut microbiota and blood immune markers of children during a 28-day biodiversity intervention. Children in standard urban and nature-oriented daycare centers were analyzed for comparison. The intervention diversified both the environmental and skin Gammaproteobacterial communities, which, in turn, were associated with increases in plasma TGF-β1 levels and the proportion of regulatory T cells. The plasma IL-10:IL-17A ratio increased among intervention children during the trial. Our findings suggest that biodiversity intervention enhances immunoregulatory pathways and provide an incentive for future prophylactic approaches to reduce the risk of immune-mediated diseases in urban societies.

## Teaser

Forest floor, sod, and gardening facilities in daycare environment modulate skin and gut microbiota and immunoregulation.

## INTRODUCTION

Observational studies have demonstrated that immune-mediated diseases are more frequent in populations adopting modern urban lifestyles than in populations with a preindustrial lifestyle (*1*–*3*). One of the leading hypotheses argues that the core reason for this pattern is the evident biodiversity loss in modern living environments (*3*–*6*). However, conclusive evidence based on human intervention trials is still lacking.

Biodiversity loss in urban areas limits exposure to diverse microbiota but increases exposure to pathogenic bacteria in densely built areas (*5*). High hygiene level and Western urban lifestyle (e.g., consumption of processed food and use of antibiotics) also influence the human commensal microbiota (*7*, *8*). Furthermore, urban pollutants alter microbial communities associated with human health and immune-mediated diseases (*9*–*11*). All of these factors may result in microbial imbalance, referred to as dysbiosis, which has been associated with immune-mediated diseases (*4*, *12*, *13*).

Early life determinants of gut microbiota include birth mode, genetics, use of antibiotics, diet, and other environmental factors (*14*). Whereas the gut microbiota of 1-year-old children are dominated by *Faecalibacterium*, *Bacteroides*, and *Anaerostipes* (*15*), healthy adult gut microbiota are characterized by the phyla Bacteroidetes and Firmicutes, particularly the genera *Bacteroides* and *Prevotella* (*16*, *17*). Lactobacillales is the dominant order on the skin of 1-year-old children, and the diversity of the skin microbiota increases with age (*18*). Core reasons why skin microbiota changes with age are related to human physiology and increased influence of external factors, such as pets and living environment (*18*). The dominant bacterial taxa on the skin of healthy adults are characterized by the phyla Actinobacteria, Firmicutes, and Proteobacteria, particularly the genera *Propionibacterium*, *Staphylococcus*, and *Corynebacterium* (*16*, *19*). Actinobacteria and Proteobacteria are also dominant phyla in soil (*5*, *6*, *10*, *11*). While skin and soil bacterial communities contain several taxa in common (*4*, *19*, *20*), bacterial taxonomies differ noticeably between soil and gut communities (*5*, *17*, *21*). Despite this taxonomic divergence, recent findings indicate that the type of ground cover and garden vegetation around permanent residences have an impact on gut microflora (*17*).

Environmental microbial exposure, human commensal microbiota, and immunological pathways are generally assumed to be interconnected (*4*, *8*, *14*, *17*). Plasma cytokine levels and blood FOXP3^+^ regulatory T (T_reg_) cell frequencies can be used as surrogates for changes in immunoregulatory pathways. Interleukin-10 (IL-10) is an anti-inflammatory cytokine, and its blood levels reflect the activation of immunoregulatory pathways (*22*). Transforming growth factor–β1 (TGF-β1) is a multifunctional cytokine that down-regulates inflammatory processes, particularly in the gut-associated immune system (*22*). IL-17 is a proinflammatory cytokine that is associated with several immune-mediated diseases, including type 1 diabetes (*23*), inflammatory bowel disease, rheumatoid arthritis, and multiple sclerosis (*24*). T_reg_ cells are essential regulators of immune system, with important roles in maintaining self-tolerance as well as tolerance to commensal microbiota, thus preventing autoimmune and chronic inflammatory diseases (*25*).

As immune-mediated diseases are an emerging health issue in urbanized societies, there is an unmet need for novel prophylactic practices to combat these maladies. To address this need, we performed an intervention study to test the biodiversity hypothesis (*3*). In this study, the environmental biodiversity of urban daycare centers was enriched by covering their yards with forest floor and sod. The effect of the intervention was studied among 75 urban children aged 3 to 5 years residing in three different daycare environments: (i) standard yards, (ii) intervention yards with biodiversity elements, and (iii) nature-oriented daycare centers where children visited nearby forests on a daily basis. We measured skin and gut microbiota, plasma cytokine levels, and blood T_reg_ frequencies in these children before and after the 28-day intervention period. Moreover, we compared environmental microbiota between the standard and intervention daycare yards. On the basis of earlier comparative studies among children (*1*, *4*), we hypothesized that the biodiversity intervention will affect the commensal microbiota of the children and that a positive change in skin microbial diversity would be associated with enhanced secretion of immunoregulatory cytokines and/or increase in T_reg_ cells after the trial. In addition, we expected the changes in commensal microbiota to reflect the composition of environmental microbiota in the intervention group and associations between the commensal microbiota and immune response to be different in this group compared to the standard daycare group.

## RESULTS

### Intervention modified surface soil microbiota in daycare yards

Ten daycare centers in two cities in Finland (Lahti and Tampere), both having populations of more than 100,000 inhabitants, were included in the study (Table 1). Three of these were nature-oriented daycare centers that served as a positive control (study subjects, *n* = 23). Each of standard urban daycare centers contained approximately 500-m^2^ yards with little or no green space. In four of these daycare yards, called “intervention daycares” hereafter, we covered part of the gravel with forest floor (100 m^2^) and sod (200 m^2^) (study subjects, *n* = 36). The three nonmodified yards (“standard daycares”) served as controls (study subjects, *n* = 16). Intervention daycares received segments of forest floor, sod, planters for growing annuals, and peat blocks for climbing and digging.

**Table 1:** Number, age, gender, and reasons for exclusion of study participants in each daycare group and in total. Each child spent daily (Monday to Friday) approximately 1.5 hours outdoors.

	Intervention* 4 centers	**Standard^†^** **3 centers**	**Nature^‡^** **3 centers**	**Total children** **10 centers**
Total children	36	16	23	75
Boys	18	9	10	37
Girls	18	7	13	38
Age^§^	4.3 ± 0.6	4.9 ± 0.3	4.7 ± 0.5	4.6 ± 0.6
Excluded^║^				
Antibiotic users	2	1	0	3
Probiotic users	1	0	1	2
Medication users	3	2	3	8

*Children in intervention daycare centers.

†Children in standard, nonmodified daycare centers.

‡Children in nature-oriented daycare centers where children visited boreal forests on a daily basis.

§Age is presented as means±SD.

║Children using probiotics, antibiotics, or medication (paracetamol, desloratadine, pyrvin, cetirizine, and salbutamol) were excluded from the gut microbiome and cytokine analyses. One child with pinworm infection was excluded from all analyses.

Vegetation in the transferred natural forest floor consisted mainly of dwarf heather (*Calluna vulgaris*), blueberries (*Vaccinium* sp.), crowberry (*Empetrum nigrum*), and mosses (*Pleurozium shreberi*, *Hylocomium splendens*, *Sphagnum* sp., and *Dicranum* sp.). The sod consisted of fescues (*Festuca* sp.) and meadow grasses (*Poa* sp.). Nurses of the daycare centers guided children to be in contact with the green materials brought into the yard. Guided activities included, for example, planting plants in planting boxes, crafting natural materials, and playing games. In addition, green materials were available to children during free outdoor activities (*26*). Children played in the yards approximately 0.5 to 2 hours twice a day in intervention and standard daycare centers (*26*). The average time outdoors was 1.5 hours.

Children were served three uniform meals daily (breakfast, lunch, and afternoon snack) in the participating daycare centers; two central kitchens, one per city, prepared the meals according to detailed guidelines for early childhood, published by Finnish food authority National Nutrition Council (www.ruokavirasto.fi/en/themes/healthy-diet/national-nutrition-council/). Private consumption of fresh plants, number of animal contacts, time spent outdoors or in nature, and number of siblings were similar among all daycare groups (table S1). The intervention lasted for 28 days in May to June 2016.

To measure differences in the environmental microbial community between modified and nonmodified daycare yards and to estimate whether the taxonomic changes were similar to those in skin bacterial communities, we compared community composition, relative abundances, richness, and diversity of bacterial communities in surface soil samples. We sampled surface soil from intervention and standard daycare yards after the intervention period of 28 days (samples collected from the sandbox and playground, next to swing, jungle gym, and the main door). The same surface soils (sandbox and playground) were sampled also on day 0 before the intervention materials arrived.

**Fig. 1:**
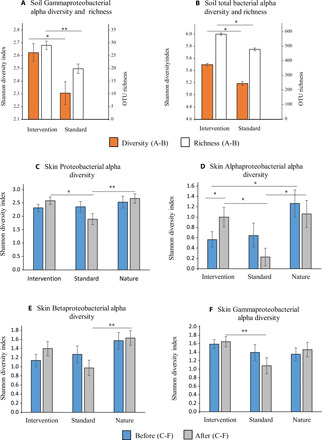
Diversity and richness of bacteria in daycare yard soils and on the skin of daycare children. After the trial, (**A**) Gammaproteobacterial and (**B**) total bacterial ground surface soil community was more diverse at intervention daycare yard soils compared to standard daycare yards. On the skin, the alpha diversity (Shannon index) of (**C**) Proteobacteria was higher among children in intervention (*n* = 29) and nature-oriented daycares (*n* = 19) compared to children in standard daycares (*n* = 13) after the study period. (**D**) Alphaproteobacterial diversity on the skin of the intervention daycare children increased during the intervention, and it was higher compared to children in standard daycares after the study period. (**E**) Betaproteobacterial diversity was higher among nature-oriented and (**F**) Gammaproteobacterial diversity higher among intervention daycare children compared to children in standard daycares after the study period. Data are displayed as means ± SE. *P < 0.05 and **P < 0.01, *t* tests after the intervention (A and B), Dunn’s multiple comparison post hoc tests after the intervention (C to E), and Wilcoxon signed-rank test (D).

In surface soil samples, the relative abundances of Proteobacteria (36% before and 34% after intervention), Bacteroidetes (24 and 28%), Actinobacteria (20 and 15%), and Acidobacteria (5 and 7%) were above 5%. After the intervention, the community compositions of these phyla and the total bacterial community differed between intervention and standard daycare yards (table S2A and fig. S1A). The intervention modified the community composition of Gammaproteobacteria in surface soils [abundance data in table S2B: permutational multivariate analysis of covariance (PERMANOVA), *P* = 0.008 and *P*_adj_ = 0.03; fig. S1B], and this community differed between the intervention and standard yards after the intervention (presence/absence data in table S2A: PERMANOVA, *P* = 0.03 and *P*_adj_ = 0.05; fig. S1B). Moreover, unclassified Gammaproteobacterial operational taxonomic units (OTUs) were enriched at intervention daycare yards, and their relative abundance was over eight times higher compared to standard daycare yards after the intervention period (table S3, A and B). Observed OTU richness and Shannon diversity of the total bacterial community (*P*_adj_ = 0.04 in both cases) and the richness of Gammaproteobacteria were higher in the intervention yards than in standard yards (Fig. 1, A and B, and table S3C). We used multivariate homogeneity of group dispersions [permutational analysis of multivariate dispersions (PERMDISP)] to examine beta diversity, and it showed that the beta diversity of five genera was higher in intervention than in standard daycare yards (table S2C).

### Intervention promoted skin bacterial diversity

Consistent with previous studies (*4*, *12*), the biodiversity intervention was associated with high diversity of skin Proteobacteria. We compared skin bacterial Shannon diversity indexes between three different daycare groups with analysis of covariance (ANCOVA) and with Kruskal-Wallis and Dunn’s multiple comparison post hoc tests using gender as a covariate. Before the intervention, children in nature-oriented daycares had divergent community composition of bacteria on skin compared to children in standard daycares (table S2D and fig. S1C) and more diverse Alphaproteobacterial skin community compared to intervention daycare children (Fig. 1D and table S4). Standard and intervention daycare groups had similar bacterial community composition (table S2D) and hosted equally diverse skin Proteobacterial communities, including classes of Alpha-, Beta-, and Gammaproteobacteria before the intervention period (Fig. 1, C to F, and table S4). After the trial, children in intervention daycares had more diverse skin Proteobacterial and Gammaproteobacterial communities than children in standard daycares (Fig. 1, C to F, and table S4). Before-after comparisons using paired *t* tests and Wilcox statistics resulted in a single difference: Alphaproteobacterial diversity increased in intervention daycares (Fig. 1D and table S5), being higher compared to children in standard daycares (Fig. 1D and table S4). These results demonstrate how the biodiversity intervention promoted or prevented the loss of skin bacterial diversity during the study period, leading to diversities comparable to those in nature-oriented daycares (Fig. 1).

### Intervention modified gut bacterial community

Because probiotics and medications, such as antibiotics, are known to affect the gut microbial community (*14*, *27*), we excluded from the gut microbial analyses those study subjects receiving probiotics or medication during the intervention (Table 1). We observed several differences associated with the daycare environment. Among children in intervention daycare centers, the relative abundance of Clostridiales decreased (*P* = 0.003 and *P*_adj_ = 0.04), and the alpha diversity of Ruminococcaceae (*P* = 0.01 and *P*_adj_ = 0.05), known to contain butyrate-producing species, increased (table S6A; paired *t* tests). No such changes were seen in children in nature-oriented or standard daycares (table S6, B and C). We additionally compared differences in community composition of gut bacteria among the three daycare groups with PERMANOVA. Children in standard and nature-oriented daycares had dissimilar community composition of Ruminococcaceae both before (*P* = 0.05 and *P*_adj_ = 0.10) and after (*P* = 0.01 and *P*_adj_ = 0.03) the intervention period, while standard and intervention daycare children had similar communities of Ruminococcaceae at the beginning of the intervention (*P* = 0.14), but slightly divergent communities at the end of the intervention (*P* = 0.03 and *P*_adj_ = 0.08; table S2E and fig. S1, E and F). Moreover, *Faecalibacterium* taxa differed in PERMANOVA between nature-oriented and standard daycare children after the intervention (*P* = 0.03; table S2F).

### Microbiota-immunological correlations

We analyzed the levels of cytokines IL-10, IL-17A, and TGF-β1 in plasma samples, and the frequency of CD4^+^CD25^+^CD127^low^FOXP3^+^ T_reg_ cells in blood samples by flow cytometry. We searched for associations between changes in plasma cytokine levels or total T_reg_ cell frequencies and changes in bacterial diversity and abundance with linear mixed-effects models (LMMs) and with nonmetric multidimensional scaling (NMDS). In addition, we performed a before-after comparison of the cytokine levels and their ratios. These comparisons demonstrated that the IL-10:IL-17A ratio increased among intervention children, but not among children in standard or nature daycares (Table 2).

**Table 2:** IL-10:IL-17A ratio before and after (mean ± SD) the 28-day intervention period in plasma of children in the intervention, standard, and nature daycares.

**Daycare center**	**Mean before**	**SD**	**Mean after**	**SD**	df*	***P***	**Adjusted *P*^†^,** **Wilcoxon paired** **test**
Intervention	0.86	0.50	1.15	0.10	21	**0.02**^‡^	**0.04**^‡^
Standard	0.93	0.47	1.04	0.44	9	0.81	1
Nature	1.22	0.92	0.96	0.55	9	0.29	0.86

*Degree of freedom. Only donors with both before and after samples were included.

†Benjamini-Hochberg correction.

‡Significant differences are indicated in boldface.

**Fig. 2:**
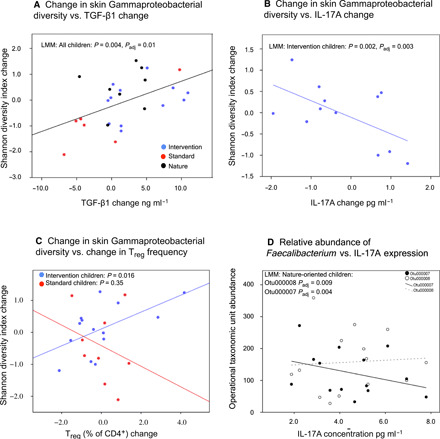
Associations between bacterial diversity and immune markers. The increase in skin Gammaproteobacterial diversity was associated (**A**) with an increase in TGF-β1 concentration in plasma (all children in an LMM) and among intervention children (**B**) with a decrease in IL-17A concentration (**C**) and increase in T_reg_ cell frequencies. In (C), the nonsignificant model for standard children is provided for comparison. (**D**) Among nature-oriented children, *F. prausnitzii* (Otu000007) was associated with a decrease and unknown *Faecalibacterium* Otu000008 with an increase in IL-17A concentration (results from end of trial).

When all children were analyzed together in an LMM, an increase in skin Gammaproteobacterial diversity was associated with an increase in TGF-β1 plasma concentration (*P*_adj_ = 0.01; Fig. 2A and table S7A), and among intervention children, this diversity increase was also associated with a decrease in IL-17A plasma level (*P*_adj_ = 0.002; Fig. 2B and table S7B) and increase in the percentage of T_reg_ cells (*P* = 0.016; Fig. 2C and table S7C). Among nature-oriented daycare children, high Gammaproteobacterial diversity in skin microbiomes was associated with an increase in IL-10 plasma concentration (*P*_adj_ = 2.3 × 10^−6^; table S7D), whereas among standard daycare children, low Gammaproteobacterial diversity was associated with a decrease in TGF-β1 expression (*P*_adj_ = 0.01; table S7E and fig. S2).

When all children were analyzed together in an NMDS model, the community composition of *Faecalibacterium* species in the gut was associated with plasma IL-17A concentration after the intervention period (*P* = 0.015 and *P*_adj_ = 0.045; table S8). Within nature-oriented children, the higher abundance of *Faecalibacterium* Otu00007 (*Faecalibacterium prausnitzii* in BLASTN; query cover = 100%, *E* value = 2 × 10^−127^, ident = 99%) was associated with lower levels of IL-17A in plasma (LMM, *P*_adj_ = 0.004), whereas a higher abundance of *Faecalibacterium* Otu000008 (no match in BLASTN with the significance threshold of 10) was associated with higher levels of IL-17A in plasma (LMM, *P*_adj_ = 0.009; Fig. 2D and table S7F). The latter association was weaker but significant also among children in standard daycares (LMM, *P*_adj_ = 0.04; table S7G). Among nature-oriented daycare children, the decrease in plasma IL-17A concentration was additionally associated with a decrease in the relative abundance of *Romboutsia* (*P*_adj_ = 5.7 × 10^−5^) and *Dorea* (*P*_adj_ = 0.03) and an increase in *Anaerostipes* (*P*_adj_ = 0.01) in the gut (table S7H).

## DISCUSSION

While early exposure to environmental biodiversity has been linked to the development of a well-functioning immune system (*1*, *4*), the final proof of causality is still lacking. This study is the first human intervention trial in which urban environmental biodiversity was manipulated to examine its effects on commensal microbiome and the immune system in young children. The 28-day-long intervention that included enrichment of daycare center yards for microbial biodiversity was associated with changes in the skin and gut microbiota of children, which, in turn, were related to changes in plasma cytokine levels and T_reg_ cell frequencies. These findings suggest that the exposure to environmental microbial diversity can change the microbiome and modulate the function of the immune system in children. Specifically, the intervention was associated with a shift toward a higher ratio between plasma cytokine IL-10 and IL-17A levels and a positive association between Gammaproteobacterial diversity and T_reg_ cell frequencies in blood, suggesting that the intervention may have stimulated immunoregulatory pathways. These regulatory changes occurred despite slightly inconsistent findings in skin microbiota (see Figs. 1 and 2). Overall, the study indicates that it may be possible to modulate the immune system by relatively simple actions that change the living environment of young children in urban communities.

The intervention maintained a high diversity of commensal skin microbiota, particularly among Gammaproteobacteria, relative to the children in standard daycares, in whom the diversity of these microbiota declined over the study period. Our results are in line with previous observational studies showing associations between immune system markers, living environment, and commensal microbiota, including skin Gammaproteobacteria (*1*, *4*, *12*). Thus, the results of the present intervention study support the biodiversity hypothesis (*3*, *4*). Because biodiversity intervention offers embodied experiences of nature and provides multisensory exploration and diverse learning situations (*26*), children might have more direct contacts with soil and vegetation in intervention than in standard daycares. Time spent in the yards might also be one explanation why the skin Proteobacterial diversities declined among children in standard daycares but not among children in intervention daycares, although time spent outdoors was similar in all daycare groups. Our conclusion is that letting urban children play in microbiologically diverse dirt and vegetation alters skin and gut microbiota, which is accompanied by parallel changes in the immune system within a relatively short period of 1 month.

An important aspect of these findings is that the commensal microbiota of children in intervention daycare centers became more similar to that observed in children attending nature-oriented daycares (Fig. 1), where children make daily visits to nearby forests. Today, a vast majority of children in developed societies, and increasing numbers in developing ones, live in urban areas (*28*), and many of them have limited access to areas characterized by rich natural biodiversity such as forests. In addition, green areas surrounding urban environments are often contaminated by pests and pathogenic microbes (*29*). For this reason, one can anticipate that providing children with a chance for daily contact with diverse vegetation and dirt in safe urban green spaces such as playgrounds and daycare center or schoolyards might improve child health by activating the regulatory pathways of the immune system. This could reduce overactive immune responses and, consequently, decrease the risk of developing immune-mediated diseases. In addition, biodiversity intervention may afford well-being benefits and increase children’s physical activity (*26*). The findings of the present study and our previous well-being study (*26*) encourage implementation of these solutions in the management and planning of the urban environments.

Exposure to microbial biodiversity affected the gut microbiota, particularly Ruminococcaceae (including *Faecalibacterium*), which includes Gram-negative bacteria associated with the maintenance of gut health (*30*). The family also contains established or candidate probiotics (*13*). The intervention increased the diversity of Ruminococcaceae, although intervention materials per se did not contain the strains found in stool samples. This raises the question of whether the mechanism of immune regulation in the biodiversity intervention was fundamentally different from the impacts of probiotic treatments; probiotics improve the abundance of certain strains only and may even delay the recovery of gut microbiota after antibiotic treatments (*31*). Probiotic treatments have also shown only limited, if any, effect in the prevention of allergies (*32*). Moreover, our approach—biodiversity intervention—exposed children to a wide spectrum of environmental microbes, including microscopic invertebrates, protozoa, fungi, archaea, and viruses. This exposure also happened via multiple routes including skin and mucosal routes. Thus, this kind of biodiversity intervention should activate a much wider spectrum of pattern recognition receptors (PRRs) than oral treatment with probiotic bacteria (*33*). However, further studies will be needed to elucidate the nature of the potential wide-spectrum PRR stimulation by the presently described approach.

The decreasing diversity of skin microbiota in the standard nonintervention daycare group may also be explained by a decrease in microbial activity during the intervention period. As the intervention started less than a month after snow melt, and as the intervention period was dry, the top of mineral soil, concrete, and asphalt plausibly dried during the intervention. Hence, it is not unexpected that Gammaproteobacterial richness and the abundance of unidentified OTUs were higher in intervention than standard yards at the end of the intervention period (see Fig. 1 and table S3). As children in the intervention group were in daily contact with rich ground surface Gammaproteobacterial community on weekdays, and as Gammaproteobacteria position themselves below subepidermal compartments in the skin (*34*), their skin Gammaproteobacterial content plausibly maintained higher compared to children in standard daycares. Touching organic landscaping materials has been shown to immediately increase the diversity of Proteobacteria, including Gammaproteobacteria, on skin (*19*). A parallel factor was the increased willingness to play with soil and plant materials in the intervention yards (*26*), leading to increased voluntary microbial exposure by children.

Standard daycare yards consisting of mostly inorganic man-made landscaping materials can be considered as man-made harsh, arid environments. As arid environments typically host low microbial activity but huge microbial diversity particularly under nutrient depletion (*35*), we were surprised to see higher Proteobacterial richness in intervention yards. It is possible that parts of the Proteobacterial community in intervention yards originated from preintervention period and that the transfer of sod and forest floor resulted in a lag time during which bacterial diversity and richness were still adapting to the new growth-supporting environment. We recommend following the effects of biodiversity intervention over a longer period to understand the microbial community dynamics on daycare yards and to see the longevity of changes in plasma cytokines.

Factors shaping immune response include changes in the external environment and exposure to human pathogens. The latter induces a proinflammatory response, e.g., affects IL-10:IL-17A ratio (see Table 2). Because heterogeneous home environments did not mask differences between daycare groups (see Fig. 1), the impact of the biodiversity intervention on commensal microbiota was strong compared to potential temporary exposures during the intervention period. As Gammaproteobacterial richness was improved and unidentified OTUs within Gammaproteobacteria were enriched at the intervention yards only, changes in skin Gammaproteobacteria in standard and nature-oriented groups were plausibly of different origin compared to the intervention group. This is not unexpected as in the standard group Gammaproteobacteria arrived from urban nongreen environment, and as children in nature-oriented daycare centers were exposed to forest biodiversity throughout the year. Therefore, and as we recently demonstrated how exposure to organic soil reduces the relative abundance of genera containing opportunistic pathogens on the skin of urban dwellers (*36*), the positive associations between skin Gammaproteobacterial diversity and plasma IL-17A levels in the nonintervention groups may be a consequence of exposure to Gammaproteobacterial pathogens or other unidentified environmental factors. In this context, it is noteworthy that the group-specific regression slopes between skin Gammaproteobacterial diversity and the multifunctional cytokine TGF-β were similar in all daycare groups and that the abundance of unidentified Faecalibacterium OTU 000008 in the gut was directly associated with plasma IL-17A in all daycare groups. *Faecalibacterium prausnitzii*, on the other hand, was associated with IL-17A in the nature-oriented group only; we recently showed that diverse vegetation, which is a proxy for diverse microbiota exposure, is associated with high *Faecalibacterium* abundance in the gut (*17*). The lack of *F. prausnitzii*–IL-17A association in standard daycare centers may result from low daily exposure to environmental biodiversity, while gut microflora in the intervention children might still be adapting to the new high-biodiversity living environment. To summarize, while associations between environmental factors and cytokines are complex, the current study highlights the profound effect of biodiversity intervention on commensal microbiota, which may explain partly divergent associations between plasma cytokines and microbiota in different daycare environments.

One of the major weaknesses of our study was the impossibility of controlling for the home environment. Families have different living environments, lifestyles, and children have varying numbers of siblings and pets. However, although families with a child in a nature-oriented daycare center may often be interested in various outdoor activities in their private life, this was not visible in the time spent outdoors or green areas. There is hardly any reason to assume an unknown bias between intervention and standard daycare centers in recruitment. Instead, we obviously cannot exclude the potential impact of a child’s behavior outside daycare centers. These uncertainties highlight the problems inherent in all attempts to perform intervention trials in a normal living environment among families having young children, but they also underline the importance of a biodiverse daycare environment and the resulting strength of associations between skin and gut microbiota and the levels of immune markers, despite the multiple confounding factors in children’s urban living environment.

In the intervention, we used slowly renewable natural forest floor, which is in limited supply. To cope with this issue, we recently proposed a wide-spectrum microbial inoculant from forest and agricultural materials that resembles microbiota in organic soils and hosts the wide spectrum of eukaryotic and prokaryotic micro-organisms described above, including hundreds of inactive and slowly growing bacterial phylotypes (*19*, *20*). In a recent study (*36*), no facultative pathogens and less opportunistic pathogens were found on human skin after contact with playground sand enriched with the wide-spectrum microbial inoculant, compared to contact with standard playground sand or no contact at all. A two-week human exposure trial (*20*) with this wide-spectrum inoculant did not lead to any negative health outcomes for the study subjects. Providing that the preliminary results can be confirmed in subsequent intervention trials, the wide-spectrum PRR stimulation by biodiversity intervention may prove to be beneficial in modulating the immune system and microbial communities of urban dwellers (*36*).

Our trial has implications on the interpretation of the results from previous observational studies (*1*–*5*, *12*). The results of this study support the biodiversity hypothesis and the concept that low biodiversity in the modern living environment may lead to an uneducated immune system and consequently increase the prevalence of immune-mediated diseases (*4*, *5*). Similarly, while earlier studies have reported a decreased *Faecalibacterium* abundance in several immune disorders (*13*, *15*), we found that a high *F. prausnitzii* abundance is associated with decreased expression of proinflammatory cytokine IL-17A among healthy children. In conclusion, our study demonstrated that modifying the living environment of children with microbiologically diverse natural materials might provide a feasible approach for decreasing the risk of immune-mediated diseases in urban populations.

## MATERIALS AND METHODS

### Study group

The study was carried out in accordance with the recommendations of the Finnish Advisory Board on Research Integrity with approval from the ethics committee of the local hospital district (Pirkanmaa Hospital District, Finland). Written informed consent obtained from all guardians was in accordance with the Declaration of Helsinki.

Subjects attending daycare centers and living in an urban environment and meeting the inclusion criteria were eligible for participation in this study. Together, 75 children, aged between 3 and 5 years, participated in the study. Exclusion criteria of the study were the following: age below 3 or over 5 at the beginning of the study, native country other than Finland, immune deficiency (e.g., antibody deficiency and HIV infection), immunosuppressive medication (e.g., corticosteroids), a condition affecting immune response (e.g., rheumatoid arthritis, colitis ulcerosa, Crohn’s disease, diabetes, and Down syndrome), or cancer diagnosis. The use of antibiotics, probiotics, medication, and other background information was recorded using standardized questionnaires.

### Sample collection

To examine the effects of intervention on commensal microbiota and immune regulation, skin, stool, and blood samples were collected from the children before the yards were transformed and after the intervention period. Ground surface soil was collected before and after the intervention period from daycare yards located in Lahti. Before the intervention, samples were collected from sand boxes and playgrounds and, after the intervention, also next to swings and under the jungle gym.

A trained study nurse collected skin swab samples in daycare centers, and the children’s parents collected stool samples at home. Skin swabs were taken from the back of the hand (5 cm–by–5 cm area), 10-s wiping with a saline buffer (0.1% Tween 20 in 0.15 M NaCl) wetted cotton wool stick. After sampling, the wool was cut to a sterile polyethene tube and frozen at −80°C until used for microbiome analyses. Stool samples were stored in home freezers (−18° to 20°C) until the researchers collected them and stored at −80°C.

A venous blood sample was taken from the arm vein into Vacutainer CPT Mononuclear Cell Preparation tubes with sodium citrate (BD Biosciences, NJ, USA) and centrifuged according to the manufacturer’s instructions to separate the plasma and peripheral blood mononuclear cells (PBMCs). PBMCs were frozen in freezing medium consisting of 10% dimethyl sulfoxide (Merck KGaA, Darmstadt, Germany), 10% human AB serum (Sigma-Aldrich, MO, USA), penicillin (50 U/ml) and streptomycin (50 μg/ml; Sigma-Aldrich, MO, USA), and 10 mM l-glutamine (Life Technologies, CA, USA) in RPMI 1640 medium (Life Technologies, CA, USA) using freezing containers at −80°C (BioCision LLC, CA, USA). The PBMC samples were transferred into liquid nitrogen for long-term storage after 48 hours. The plasma samples were stored at −80°C.

### Sample preparation for MiSeq sequencing and bioinformatics

Skin and stool samples for MiSeq sequencing were prepared as in the study of Roslund *et al.* (*11*). Soil, skin, and stool samples were sequenced using Illumina MiSeq 16*S* ribosomal RNA (rRNA) gene metabarcoding with read length 2 × 300 base pairs using a v3 reagent kit. Raw data were processed in Mothur (version 1.39.5) (*37*), as described by Roslund *et al.* (*10*).

Bacterial sequences were aligned against a SILVA reference (version 123) and classified using the Mothur version of Bayesian classifier with the RDP training set version 16 with 80% bootstrap threshold. Low-abundance OTUs (≤10) and OTUs detected in negative extraction and polymerase chain reaction (PCR) controls were removed from sequence data. There were 3 OTUs detected in skin, 0 OTUs in stool, and 21 OTUs (3 is the same that in skin) detected in soil DNA extraction or PCR control (Sterile water). Observed OTU richness and the Shannon diversity were calculated in Mothur. Soil and stool samples were subsampled to 3842 and skin samples to 329 sequence depth for bacterial community composition analyses. For skin samples, 13 of the 28-day samples were not collected or the number of sequences was too low, and thus, 29 children from intervention daycares, 19 from nature-oriented daycares, and 13 from standard daycares were included in skin sample analyses. Good’s coverage index (average ± SD: soil, 0.96 ± 0.02; stool, 1.00 ± 0.00; skin, 0.96 ± 0.06) was used to determine OTU coverage adequacy for diversity and community composition analyses. Because statistical tests showed associations within the genus *Faecalibacterium*, OTUs of *Faecalibacterium* were further identified with microbe BLASTN (version 2.8.1+).

### Cytokine and T_reg_ cell analyses

IL-17A and IL-10 concentrations were measured from plasma samples using a Milliplex MAP high-sensitivity T cell panel kit (Merck KGaA, Darmstadt, Germany) according to the manufacturer’s instruction. Fluorescence was analyzed using a Bio-Plex 200 system (Bio-Rad Laboratories, Hercules, CA, USA), and data were collected using Bio-Plex Manager software (version 4.1, Bio-Rad Laboratories, Hercules, CA, USA). TGF-β1 concentration was analyzed using enzyme-linked immunosorbent assay (Bender MedSystems GmbH, Vienna, Austria).

Frozen PBMCs were thawed, and immunostaining to identify CD3^+^CD4^+^CD25^+^CD127^low^FOXP3^+^ T_reg_ cells was performed as previously described (*38*). The frequency of T_reg_ cells was defined as a percentage of total CD3^+^CD4^+^ T cells.

### Statistical analyses

All statistical tests were done in R version 3.4.2 (R Development Core Team, 2018) (*39*) with two-sided tests. Differences in community composition were analyzed with PERMANOVA (function adonis in vegan package) (*40*) with Bray-Curtis metric. PERMANOVA was analyzed with bacterial abundance and presence-absence datasets. PERMANOVA was done at OTU, genus, order, family, class, and phylum level and within some specific taxonomy if the relative abundance was more than 5% (skin and stool samples) and whenever there was a reason to assume biological relevance. In the data from stool samples, we analyzed differences in the compositional centroids of *Faecalibacterium* OTUs, as comparative studies between patients with immune system disorders and healthy individuals have reported decreased abundance of *Faecalibacterium* in the gut of the patients (*13*, *15*), and because the abundance of this clade in our data exceeded 5% data. NMDS was used with the Bray-Curtis metric to score the *Faecalibacterium* OTUs onto an ordination, and correlation with corresponding cytokine expression levels was assessed using the envit function in the vegan package in R. In skin samples, we analyzed Gammaproteobacterial communities because they have been connected to the living environment, expression of IL-10, and incidence of atopy (*4*). In stool and skin samples, we also analyzed taxa at the phylum, class, order, or family level if the abundance was more than 5%. As the abundance and diversity of bacteria in particularly organic soils is extremely high (*21*), for soil samples from the daycare yards, we analyzed taxa at the phylum, class, order, family, and genus levels with PERMANOVA if the abundance was more than 1%.

The Shannon diversity index was used to assess alpha diversity, and multivariate homogeneity of group dispersions (PERMDISP) was used to examine beta diversity. Differences in bacterial richness (Chao index, rarefied species, and observed OTU richness), alpha diversity, and relative bacterial abundances between daycare children were determined using ANCOVA or, in case of nonnormally distributed data, with the Kruskal-Wallis test. Gender was used as a covariate. Pairwise comparison was done with Tukey’s honestly significant difference (HSD) test or with Dunn’s test. The association between bacterial diversity and abundance changes, and cytokine expression levels and changes were evaluated using LMMs (function lmer in lme4 package). LMMs were constructed for bacterial taxonomy with observed changes, i.e., relative abundance, diversity, and richness changes during the intervention. In LMMs, cytokine expression or T_reg_ cell level change was used as a dependent variable, bacterial changes as explanatory variables, and daycare center as a random variable.

Differences between the time points were determined using the paired *t* test or, in case of nonnormally distributed data, with the Wilcoxon signed-rank test. To conceptualize the false discovery rate, all the statistical tests were carried out with Benjamini-Hochberg correction (referred to as *P*_adj_ in Results).

Primary population for the analysis was intention to treat (ITT). Per protocol (PP) (complete cases) population was used when changes between two time points (baseline and day 28) were analyzed. In addition, PP population for other analyses was used as a sensitivity analysis to investigate whether conclusions are sensitive to assumptions regarding the pattern of missing data. These sensitivity analyses were done for significant results, i.e., skin and cytokine ANCOVA, and Ruminococcaceae and *Faecalibacterium* PERMANOVA.

The primary outcome measure for the power calculation is the difference between intervention and standard control daycare study subjects in the change of Gammaproteobacterial diversity on the skin between baseline and day 28. We used prior effect estimates from the previous study that estimate correlations between environmental biodiversity, human microbiota, and immune function (*4*).

## Supplementary Material

aba2578_SM.pdf

## SUPPLEMENTARY MATERIALS

Supplementary material for this article is available at http://advances.sciencemag.org/cgi/content/full/6/42/eaba2578/DC1

## Appendix

View/request a protocol for this paper from *Bio-protocol*.
