# Supplementary material for: Biodiversity intervention enhances immune regulation and health-associated commensal microbiota among daycare children
Source: Sci Adv. 2020 Oct 14;6(42):eaba2578. doi: 10.1126/sciadv.aba2578 (PMC7556828; doi:10.1126/sciadv.aba2578)
Supplement: aba2578_SM.pdf [file aba2578_SM.pdf]

Science Advances
AAAS

advances.sciencemag.org/cgi/content/full/6/42/eaba2578/DC1

Supplementary Materials for

Biodiversity intervention enhances immune regulation and health-associated commensal microbiota among daycare children

Marja I. Roslund, Riikka Puhakka, Mira Grönroos, Noora Nurminen, Sami Oikarinen, Ahmad M. Gazali, Ondřej Cinek, Lenka Kramná, Nathan Siter, Heli K. Vari, Laura Soininen, Anirudra Parajuli, Juho Rajaniemi, Tuure Kinnunen, Olli H. Laitinen, Heikki Hyöty, Aki Sinkkonen*, ADELE research group

*Corresponding author. Email: aki.sinkkonen@luke.fi

Published 14 October 2020, *Sci. Adv.* **6**, eaba2578 (2020)
DOI: 10.1126/sciadv.aba2578

This PDF file includes:

Tables S1 to S8
Figs. S1 and S2

Table S1. Characteristics of study participants.

Kruskal-Wallis tests showed that there are no differences in days spent in daycare centers or summer cottage, sick days, outdoor recreation (Home yard, neighborhood or nature), animal contact, nutrition (berries and vegetables) and number of siblings between children in intervention, standard and nature-oriented daycares.

Days spend in daycares, summer cottage, and siblings and sick days during the intervention period are presented as mean ± standard deviation (sd).

Outdoor recreation and animal contact during the intervention period are presented at nominal scale (median ± confidence level 95% = cl):

1 = not at all, 2 = rarely, 3 = weekly, 4 = almost daily and 5 = daily.

Nutrition is presented at nominal scale (median ± confidence level 95% = cl):

1 = not at all, 2 = occasionally, 3 = frequently.

	**Intervention**		**Standard**		**Nature-oriented**		**Kruskal-Wallis test**	
	**Mean**	**sd**	**Mean**	**sd**	**Mean**	**sd**	**Chi-squared**	**P value**
Daycare days	15.800	3.398	14.333	2.664	14.864	3.468	2.169	0.338
Sick days	0.567	0.898	1.233	2.499	0.591	0.908	0.086	0.958
Days in summer cottage	0.967	1.866	0.600	0.910	1.364	1.814	2.176	0.337
Siblings	1.133	0.937	0.867	0.834	1.455	0.963	3.768	0.152
	**Median**	**cl**	**Median**	**cl**	**Median**	**cl**	**Chi-squared**	**P value**
Outdoor recreation home yard	4	0.431	4	0.700	5	0.265	4.166	0.125
Outdoor recreation surrounding neighbourhood	4	0.300	4	0.478	3	0.333	4.822	0.090
Outdoor recreation nature	3	0.305	2	0.412	2.5	0.380	1.543	0.462
Animal contact	2	0.261	2	0.532	2	0.237	2.605	0.272
Nutrition berries	2	0.194	2	0.310	2	0.202	0.160	0.923
Nutrition vegetables	2	0.300	2	0.390	2	0.271	1.533	0.465

Table S2. Bacterial community composition (PERMANOVA) comparisons in soil (A-C), on skin (D) and in the gut (E and F) between daycare groups. Differences in surface soil bacterial communities (**A**) between intervention and standard daycare centers after the intervention and (**B**) before-after comparisons at class level. (**C**) Comparison of beta-diversity of surface soil bacterial communities between intervention and standard (PERMDISP) daycare soils at genus level after the intervention period. Community composition of bacteria (**D**) on skin at OTU level differed between children in nature-oriented and children in standard daycares at baseline. (**E**) Gut Ruminococcaceae community differed between nature-oriented and standard at baseline, and after intervention period also between children in intervention and standard daycares. (**F**) *Faecalibacterium* community composition in the gut differed between children in nature-oriented and standard daycares after the intervention period. The leftmost panel indicates groups and time points compared. Both relative abundance (left) and presence absence data (right) were compared. PERMANOVA statistics are reported as F statistics, R2 = Coefficient of determination, probability P value and Benjamini-Hochberg adjusted P value and PERMDISP results as F statistics, mean ± sd, P value and Benjamini-Hochberg adjusted P value.

	ABUNDANCE DATA				PRESENCE/ABSENCE DATA			
**A) Soil: Intervention vs Standard**	**F value**	**R2**	**P value**	**P adjust***	**F value**	**R2**	**P value**	**P adjust***
OTU level	2.053	0.079	**0.024**	**0.048**	2.226	0.085	**0.017**	0.102
Genus level	2.368	0.090	**0.023**	**0.048**	1.729	0.067	0.083	0.166
Family level	2.500	0.094	**0.021**	**0.048**	1.486	0.058	0.163	0.244
Order level	2.126	0.081	**0.049**	**0.074**	1.321	0.052	0.244	0.244
Class level	1.840	0.071	0.072	0.086	1.476	0.058	0.227	0.244
Phylum level	1.945	0.075	0.101	0.101	2.827	0.105	0.059	0.166
Phylum Proteobacteria	2.179	0.083	**0.018**	**0.030**	2.083	0.080	**0.022**	**0.037**
Phylum Bacteroidetes	2.014	0.077	**0.011**	**0.030**	2.393	0.091	**0.017**	**0.037**
Phylum Actinobacteria	2.240	0.085	**0.014**	**0.030**	2.720	0.102	**0.015**	**0.037**
Phylum Acidobacteria	2.016	0.077	**0.024**	**0.030**	1.991	0.077	**0.039**	**0.049**
Phylum Verrucomicrobia	1.544	0.060	0.084	0.084	1.615	0.063	0.111	0.111
Class Actinobacteria	2.229	0.085	**0.02**	0.055	2.693	0.101	**0.011**	**0.044**
Class Sphingobacteriia	1.874	0.072	**0.038**	0.084	2.391	0.091	**0.021**	**0.046**
Class Alphaproteobacteria	2.291	0.087	**0.015**	0.055	2.232	0.085	**0.025**	**0.046**
Class Betaproteobacteria	1.945	0.075	0.102	0.121	2.827	0.105	**0.047**	0.054
Class Gammaproteobacteria	1.497	0.059	0.138	0.138	2.159	0.083	**0.025**	**0.046**
Class Flavobacteriia	1.945	0.075	0.101	0.121	2.827	0.105	0.056	0.056
Class Cytophagia	2.512	0.095	**0.007**	0.055	2.515	0.095	**0.012**	**0.044**
Class Acidobacteria Gp4	1.945	0.075	0.082	0.121	2.827	0.105	**0.047**	0.054
Class Deltaproteobacteria	1.794	0.070	**0.01**	0.055	2.059	0.079	**0.011**	**0.044**
Class Spartobacteria	1.945	0.075	0.089	0.121	2.827	0.105	**0.044**	0.054

Class Gemmatimonadetes	1.945	0.075	0.11	0.121	2.827	0.105	**0.049**	0.054
Order Actinomycetales	2.202	0.084	**0.021**	0.063	2.763	0.103	**0.008**	**0.038**
Order Sphingobacteriales	1.874	0.072	**0.037**	0.067	2.391	0.091	**0.015**	**0.038**
Order Flavobacteriales	0.987	0.040	0.456	0.497	1.704	0.066	0.073	0.110
Order Cytophagales	2.512	0.095	**0.006**	0.063	2.515	0.095	**0.016**	**0.038**
Order Xanthomonadales	1.627	0.063	0.075	0.090	2.303	0.088	**0.030**	0.051
Order Rhizobiales	2.398	0.091	**0.013**	0.063	2.292	0.087	**0.012**	**0.038**
Order Rhodospirillales	2.395	0.091	**0.017**	0.063	2.985	0.111	**0.007**	**0.038**
Order Spartobacteria unclassified	1.829	0.071	**0.039**	0.067	2.356	0.089	**0.024**	**0.048**
	**ABUNDANCE DATA**				**PRESENCE/ABSENCE DATA**			
**B) Soil: Intervention before vs after**	**F value**	**R2**	**P value**	**P adjust***	**F value**	**R2**	**P value**	**P adjust***
Class Actinobacteria	2.319	0.072	**0.011**	**0.030**	2.243	0.070	**0.016**	0.059
Class Sphingobacteriia	2.255	0.070	**0.011**	**0.030**	2.037	0.064	**0.043**	0.079
Class Alphaproteobacteria	2.073	0.065	**0.018**	**0.040**	1.872	0.059	**0.035**	0.077
Class Betaproteobacteria	1.603	0.051	0.153	0.153	2.046	0.064	0.133	0.147
Class Gammaproteobacteria	2.250	0.070	**0.008**	**0.030**	2.329	0.072	**0.011**	0.059
Class Flavobacteriia	1.603	0.051	0.148	0.153	2.046	0.064	0.132	0.147
Class Cytophagia	1.706	0.054	**0.042**	0.077	2.128	0.066	**0.025**	0.069
Class Acidobacteria Gp4	1.603	0.051	0.143	0.153	2.046	0.064	0.147	0.147
Class Deltaproteobacteria	2.059	0.064	**0.002**	**0.022**	2.741	0.084	**0.001**	**0.011**
Class Spartobacteria	1.603	0.051	0.137	0.153	2.046	0.064	0.147	0.147
Class Gemmatimonadetes	1.603	0.051	0.149	0.153	2.046	0.064	0.136	0.147

**C) PERMDISP**	**ABUNDANCE DATA**							**PRESENCE/ABSENCE DATA**						
**SOIL**		**Standard**		**Intervention**					**Standard**		**Intervention**			
**Intervention vs Standard**	**F**	**Mean**	**sd**	**Mean**	**sd**	**P value**	**P adjust***	**F**	**Mean**	**sd**	**Mean**	**sd**	**P value**	**P adjust***
Actinomycetales unclassified	23.2	0.6	0.1	0.4	0.1	**0.001**	**0.026**	28.7	0.6	0.1	0.3	0.1	**0.001**	**0.026**
Hymenobacter	12.7	0.5	0.2	0.3	0.1	**0.003**	**0.035**	6.5	0.5	0.2	0.3	0.1	**0.008**	0.069
Noviherbaspirillum	13.6	0.5	0.2	0.2	0.1	**0.004**	**0.035**	0.1	0.1	0.3	0.1	0.2	0.718	0.849
Sphingomonas	7.5	0.5	0.2	0.3	0.1	**0.009**	**0.047**	3.4	0.4	0.2	0.3	0.1	0.076	0.395
Marmoricola	6.7	0.4	0.2	0.2	0.2	**0.009**	**0.047**	0.1	0.1	0.2	0.1	0.2	0.806	0.873
Massilia	6.1	0.4	0.2	0.3	0.1	**0.016**	0.069	0.6	0.2	0.2	0.2	0.1	0.54	0.78
Chitinophagaceae unclassified	1.4	0.5	0.1	0.5	0.2	0.277	0.463	1.4	0.5	0.2	0.4	0.1	0.262	0.56
Nocardioides	1.3	0.5	0.2	0.4	0.2	0.282	0.463	0.5	0.4	0.2	0.3	0.1	0.489	0.748
Arthrobacter	1.8	0.4	0.2	0.3	0.2	0.198	0.463	1.9	0.2	0.1	0.2	0.2	0.186	0.56
Intrasporangiaceae unclassified	1.5	0.4	0.3	0.3	0.2	0.223	0.463	1.3	0.3	0.2	0.2	0.1	0.276	0.56
Halomonas	1.5	0.4	0.3	0.3	0.2	0.214	0.463	1.3	0.3	0.2	0.2	0.1	0.28	0.56
Chryseobacterium	1.2	0.6	0.2	0.6	0.2	0.307	0.463	0.2	0.4	0.2	0.4	0.3	0.682	0.849
Burkholderiales unclassified	1.2	0.4	0.2	0.3	0.2	0.298	0.463	5.1	0.4	0.1	0.3	0.2	0.032	0.208
Xanthomonadaceae unclassified	1.7	0.5	0.2	0.6	0.2	0.201	0.463	0	0.5	0.1	0.5	0.2	0.989	0.989
Microbacteriaceae unclassified	0.9	0.4	0.2	0.3	0.1	0.338	0.463	15.6	0.4	0.1	0.3	0.1	0.004	0.052
Sphingomonadaceae unclassified	1.1	0.6	0.1	0.6	0.2	0.305	0.463	1.4	0.6	0.2	0.5	0.5	0.249	0.56
Betaproteobacteria unclassified	2.5	0.6	0.1	0.6	0.2	0.135	0.463	1.0	0.5	0.2	0.5	0.2	0.353	0.612
Comamonadaceae unclassified	0.7	0.5	0.2	0.4	0.2	0.396	0.515	0.7	0.3	0.2	0.3	0.2	0.404	0.657

Pseudomonas	0.5	0.5	0.2	0.6	0.2	0.488	0.604	0.3	0.3	0.3	0.4	0.4	0.605	0.828
Pedobacter	0.1	0.5	0.1	0.5	0.1	0.7	0.827	1.6	0.4	0.2	0.3	0.1	0.215	0.56
Ferruginibacter	0.1	0.6	0.1	0.6	0.2	0.757	0.856	0.1	0.5	0.2	0.4	0.2	0.801	0.873
Flavobacterium	0.0	0.5	0.2	0.5	0.2	0.851	0.899	0.8	0.5	0.2	0.4	0.2	0.352	0.612
Spartobacteria unclassified	0.0	0.6	0.1	0.6	0.2	0.864	0.899	1.5	0.5	0.1	0.5	0.2	0.262	0.56

**D) Skin: OTU level. baseline**	**Abundance data**			**Presence/absence data**			
	**F value**	**R2**	**P value**	**F value**	**R2**	**P value**	**P adjust**
Intervention vs standard	0.986	0.021	0.456	0.946	0.020	0.555	0.555
Intervention vs nature-oriented	1.321	0.025	0.060	1.388	0.026	0.070	0.105
Nature-oriented vs standard	1.241	0.040	0.102	1.474	0.047	**0.025**	0.075

**E) Gut: Family Ruminococcaceae**	**Abundance data**				**Presence/absence data**			
	**F value**	**R2**	**P value**	**P adjust***	**F value**	**R2**	**P value**	**P adjust***
Intervention before vs after	0.528	0.010	0.871		0.389	0.007	0.919	
Standard before vs after	0.530	0.021	0.895		0.532	0.021	0.808	
Nature before vs after	0.174	0.005	0.997		0.357	0.010	0.903	
Nature vs Standard before	1.928	0.057	**0.047**	0.101	2.448	0.071	**0.020**	0.079
Interventio vs Standard before	1.473	0.036	0.143		1.052	0.026	0.409	
Nature vs Intervention before	0.511	0.011	0.881		1.757	0.037	0.093	
Nature vs Standard after	2.265	0.070	**0.010**	**0.030**	1.222	0.039	0.301	
Intervention vs Standard after	1.942	0.049	**0.027**	0.081	1.463	0.037	0.163	
Nature vs Intervention after	0.644	0.014	0.793		0.613	0.014	0.777	

**F) Gut: Genus Faecalibacterium****	**Abundance data**			**Presence/absence data**		
	**F value**	**R2**	**P value**	**F value**	**R2**	**P value**
Intervention before vs after	0.158	0.003	0.943	-0.027	-0.001	0.876
Standard before vs after	1.293	0.049	0.261	1.122	0.043	0.474
Nature before vs after	0.310	0.008	0.813	0.543	0.014	0.792
Nature vs Standard before	1.705	0.051	0.185	1.554	0.046	0.272
Interventio vs Standard before	1.705	0.041	0.136	1.362	0.033	0.317
Nature vs Intervention before	0.492	0.011	0.770	-0.692	-0.015	0.968
Nature vs Standard after	3.233	0.097	**0.030**	0.150	0.005	1.000
Intervention vs Standard after	2.288	0.057	0.086	0.240	0.006	0.795
Nature vs Intervention after	0.669	0.015	0.592	0.116	0.003	0.950

*Benjamini-Hochberg correction

***Faecalibacterium* was the sole genus analyzed

Table S3. Comparison of bacterial **(A)** relative abundance at genus level between intervention and standard daycare center surface soils and **(B)** between baseline and day-28 at intervention daycare yards (Wilcoxon signed-rank test). **(C)** Comparison of bacterial richness and diversity between intervention and standard daycare center surface soils at total bacterial and class levels (t-test). Data is shown as mean ± standard deviation = sd.

**A) Intervention vs standard, Degrees of Freedom = 23**	**Mean standard**	**sd**	**Mean intervention**	**sd**	**Statistic**	**P value**	**P adjust**
**Flavobacterium**	134.3	102.0	233.0	168.4	108.0	0.147	0.491
**Chitinophagaceae unclassified**	261.1	123.6	202.1	72.9	51.5	0.140	0.484
**Sphingomonas**	208.6	47.3	159.8	108.3	50.5	0.126	0.484
**Massilia**	231.4	96.9	141.9	114.9	45.0	0.069	0.365
**Hymenobacter**	193.7	76.1	114.9	125.2	45.0	0.069	0.365
**Ferruginibacter**	185.9	110.5	112.4	82.2	46.0	0.077	0.367
**Mucilaginibacter**	18.3	28.3	94.6	164.6	106.0	0.178	0.494
**Pedobacter**	82.4	62.3	90.4	83.2	82.0	0.937	1.000
**Spartobacteria unclassified**	22.7	18.0	81.9	90.5	118.0	0.048	0.300
**Nocardioides**	84.6	37.3	76.1	51.6	74.0	0.772	0.963
**Gammaproteobacteria unclassified**	8.3	6.1	64.7	77.2	137.0	**0.003**	0.052
**Arthrobacter**	71.3	61.0	63.3	79.4	58.0	0.257	0.578
**Betaproteobacteria unclassified**	28.6	21.9	61.8	92.7	81.5	0.958	1.000
**Burkholderiales unclassified**	58.3	23.6	61.0	33.0	77.0	0.895	1.000
**Chryseobacterium**	122.1	263.5	55.1	76.2	87.5	0.712	0.942
**Intrasporangiaceae unclassified**	63.9	27.3	50.6	30.2	60.5	0.317	0.678
**Xanthomonadaceae unclassified**	47.7	69.6	49.8	37.3	103.5	0.225	0.563
**Microbacteriaceae unclassified**	43.1	19.1	47.4	36.5	78.5	0.958	1.000
**Comamonadaceae unclassified**	48.4	17.0	45.5	23.4	77.0	0.895	1.000
**Actinomycetales unclassified**	46.5	22.6	40.6	36.9	51.5	0.140	0.484
**Luteolibacter**	27.4	23.3	40.1	39.0	91.5	0.561	0.901
**Cryobacterium**	40.8	26.7	39.8	45.8	63.0	0.384	0.780
**Aridibacter unclassified**	50.8	30.4	39.7	35.8	63.5	0.399	0.780
**Sphingomonadaceae unclassified**	47.4	23.1	39.0	18.9	66.0	0.476	0.874
**Myxococcales unclassified**	25.4	21.8	35.8	35.7	89.0	0.654	0.942
**Gp6 unclassified**	17.8	12.3	35.6	55.8	80.0	1.000	1.000
**Oxalobacteraceae unclassified**	46.4	27.0	34.4	26.6	58.0	0.257	0.578
**Mycobacterium**	7.1	4.7	33.8	31.4	140.5	0.002	0.052
**Bacteroidetes unclassified**	21.6	20.5	33.1	28.4	102.5	0.246	0.578
**Granulicella unclassified**	6.7	16.5	32.9	76.1	79.0	0.975	1.000
**Clostridium sensu stricto**	5.7	4.9	32.9	56.0	120.5	**0.035**	0.240
**Bradyrhizobium**	14.0	8.6	32.8	22.1	121.0	**0.033**	0.240
**Acidobacteria Gp1 unclassified**	2.8	3.1	31.4	63.3	74.0	0.762	0.963
**Marmoricola**	49.5	15.0	30.1	27.6	38.5	**0.031**	0.240
**Gemmatimonas**	56.0	42.5	30.0	30.6	53.5	0.170	0.494
**Noviherbaspirillum**	80.0	34.3	29.9	32.7	20.5	**0.002**	0.052
**Rhizobiales unclassified**	24.5	18.0	28.5	26.2	74.5	0.792	0.963
**Burkholderia**	4.0	6.1	26.2	53.7	92.0	0.537	0.901

**Polaromonas**	22.3	20.7	24.8	21.1	85.5	0.792	0.963
**Alphaproteobacteria unclassified**	5.9	6.0	24.5	31.3	110.0	0.119	0.484
**Novosphingobium**	24.0	15.3	23.7	23.3	70.5	0.635	0.942
**Spirosoma**	46.0	31.2	23.6	25.8	50.5	0.124	0.484
**Gp3 unclassified**	12.1	10.7	23.4	28.6	88.5	0.672	0.942
**Pseudomonas**	27.7	30.7	22.9	30.9	68.0	0.544	0.901
**Actinobacteria unclassified**	11.4	8.5	22.7	16.0	117.5	**0.050**	0.300
**Arenimonas**	26.0	18.3	22.6	22.1	71.5	0.672	0.942
**Sinobacteraceae unclassified**	14.4	19.3	21.9	28.6	89.0	0.653	0.942
**Planctomycetaceae unclassified**	4.2	4.4	20.6	19.6	123.0	0.025	0.227
**Brevundimonas**	15.2	29.2	20.2	23.0	100.0	0.303	0.665
**Proteobacteria unclassified**	19.0	7.2	20.1	17.9	73.5	0.751	0.963
**Gaiella**	8.5	6.2	18.3	29.2	79.0	0.979	1.000
**candidate division WPS.1 unclassified**	8.7	10.0	18.0	17.8	106.5	0.169	0.494
**Acidobacteria Gp4 unclassified**	23.8	16.7	17.9	15.5	64.0	0.413	0.791
**Blastocatella unclassified**	20.1	8.0	16.8	35.2	33.0	**0.014**	0.159
**Duganella**	21.4	14.3	16.7	15.7	58.0	0.256	0.578
**Terrimonas**	7.7	6.1	16.6	19.4	96.5	0.398	0.780
**Nakamurella**	19.2	13.8	16.5	9.2	77.5	0.916	1.000
**Subdivision3 unclassified**	6.7	7.2	15.4	18.5	93.0	0.501	0.874
**Opitutus**	6.3	6.1	14.9	21.0	98.5	0.340	0.712
**Cytophagales unclassified**	16.1	10.9	14.6	20.2	54.0	0.178	0.494
**Roseomonas**	24.5	13.9	14.4	14.7	47.5	0.091	0.410
**Acidobacteria Gp3 unclassified**	0.8	8.8	14.1	20.5	76.0	0.853	1.000
**Dyadobacter**	10.1	15.1	13.7	18.6	93.5	0.489	0.874
**Cellvibrio**	20.4	29.9	13.6	12.7	78.0	0.937	1.000
**Acetobacteraceae unclassified**	35.4	58.8	13.6	20.7	55.5	0.205	0.526
**Gp4 unclassified**	4.6	8.2	13.3	40.4	76.5	0.869	1.000
**Rhizobium**	11.2	6.0	12.2	9.7	78.0	0.937	1.000
**Rhodopseudomonas**	17.6	7.0	12.1	10.4	51.5	0.140	0.484
**Nocardioidaceae unclassified**	10.9	9.8	12.1	10.2	80.5	1.000	1.000
**Flavisolibacter**	38.3	21.4	12.0	13.7	22.5	**0.003**	0.052
**Caulobacter**	11.3	6.4	11.5	9.7	78.0	0.937	1.000
**Conexibacter**	8.2	8.6	11.1	15.1	72.0	0.690	0.942
**Chryseolinea unclassified**	7.8	7.5	11.1	12.3	89.5	0.634	0.942
**Roseiarcus**	0.1	0.3	10.4	23.7	99.0	0.187	0.494
**Pseudoxanthomonas**	1.5	2.3	10.0	8.6	134.5	**0.004**	0.052
**Deltaproteobacteria unclassified**	7.8	5.2	9.9	13.2	73.5	0.751	0.963
**Cellulomonas**	6.5	5.3	8.6	6.9	91.5	0.560	0.901
**Phenylobacterium**	7.5	5.9	8.1	9.6	72.0	0.691	0.942
**Chloroflexi unclassified**	2.6	2.9	7.5	8.5	105.5	0.183	0.494
**Micromonosporaceae unclassified**	17.0	9.2	7.5	9.4	24.0	**0.003**	0.052
**Actinoplanes**	5.2	5.7	7.2	14.6	67.0	0.505	0.874
**Gp7 unclassified**	5.0	7.6	7.1	19.0	70.0	0.592	0.935
**Hyphomicrobium**	2.0	1.8	6.6	10.7	112.0	0.076	0.367
**Cytophaga**	9.9	14.6	6.5	10.4	72.5	0.708	0.942

**Segetibacter**	33.6	30.8	5.8	7.9	25.0	**0.004**	0.052
**Moraxellaceae unclassified**	3.1	6.6	5.7	14.7	87.5	0.674	0.942
**Sphingomonadales unclassified**	14.4	9.7	5.7	7.7	37.5	0.025	0.227
**Rhizomicrobium unclassified**	0.0	0.0	5.3	11.7	95.0	0.169	0.494
**Solirubrobacterales unclassified**	9.0	11.3	5.2	7.1	66.0	0.473	0.874
**Solirubrobacter**	2.8	2.6	5.1	7.9	78.0	0.935	1.000
**B) Intervention yards baseline vs day-28, Degrees of Freedom = 31**	**Mean baseline**	**sd**	**Mean day-28**	**sd**	**Statistic**	**P value**	**P adjust**
**Flavobacterium**	171.8	145.6	233.0	168.4	80.0	0.552	0.869
**Chitinophagaceae unclassified**	213.6	72.3	202.1	72.9	48.5	0.326	0.606
**Bacteria unclassified**	206.0	157.4	180.2	66.4	57.0	0.587	0.870
**Sphingomonas**	155.8	77.2	159.8	108.3	73.0	0.816	0.970
**Massilia**	86.9	46.8	141.9	114.9	91.0	0.245	0.584
**Hymenobacter**	100.1	72.5	114.9	125.2	68.0	1.000	1.000
**Ferruginibacter**	123.7	69.6	112.4	82.2	60.0	0.698	0.933
**Mucilaginibacter**	7.9	9.1	94.6	164.6	102.0	**0.018**	0.279
**Pedobacter**	65.1	44.7	90.4	83.2	70.5	0.570	0.869
**Spartobacteria unclassified**	27.3	53.0	81.9	90.5	111.0	**0.028**	0.280
**Nocardioides**	123.5	60.6	76.1	51.6	24.0	**0.024**	0.279
**Gammaproteobacteria unclassified**	14.9	12.3	64.7	77.2	109.0	**0.036**	0.290
**Arthrobacter**	89.4	67.4	63.3	79.4	43.0	0.205	0.566
**Betaproteobacteria unclassified**	30.2	40.5	61.8	92.7	83.0	0.453	0.740
**Burkholderiales unclassified**	56.1	29.7	61.0	33.0	64.5	0.877	0.970
**Chryseobacterium**	34.9	60.9	55.1	76.2	95.5	0.162	0.500
**Intrasporangiaceae unclassified**	98.3	70.3	50.6	30.2	26.0	**0.032**	0.283
**Xanthomonadaceae unclassified**	65.5	45.2	49.8	37.3	46.5	0.277	0.584
**Microbacteriaceae unclassified**	44.3	36.7	47.4	36.5	79.5	0.569	0.869
**Comamonadaceae unclassified**	45.6	20.8	45.5	23.4	68.5	1.000	1.000
**Actinomycetales unclassified**	53.3	64.1	40.6	36.9	62.5	0.796	0.970
**Luteolibacter**	24.9	21.6	40.1	39.0	91.5	0.234	0.584
**Cryobacterium**	28.4	30.7	39.8	45.8	72.0	0.856	0.970
**Aridibacter unclassified**	44.0	24.2	39.7	35.8	55.5	0.820	0.970
**Sphingomonadaceae unclassified**	48.6	25.0	39.0	18.9	50.5	0.379	0.632
**Myxococcales unclassified**	22.3	23.5	35.8	35.7	78.5	0.306	0.584
**Gp6 unclassified**	21.4	18.3	35.6	55.8	66.0	0.938	0.988
**Oxalobacteraceae unclassified**	25.7	18.6	34.4	26.6	93.5	0.196	0.560
**Mycobacterium**	8.1	5.0	33.8	31.4	129.0	**0.002**	0.114
**Bacteroidetes unclassified**	39.9	24.2	33.1	28.4	45.5	0.255	0.584
**Granulicella unclassified**	0.8	4.6	32.9	76.1	18.0	0.142	0.455
**Clostridium sensu stricto**	11.6	20.0	32.9	56.0	84.0	0.052	0.326
**Bradyrhizobium**	16.0	10.1	32.8	22.1	102.5	0.079	0.370
**Acidobacteria Gp1 unclassified**	12.1	46.4	31.4	63.3	46.0	0.264	0.584
**Marmoricola**	73.8	41.7	30.1	27.6	7.0	**0.003**	0.114
**Gemmatimonas**	43.3	25.1	30.0	30.6	50.0	0.365	0.632
**Noviherbaspirillum**	57.7	85.2	29.9	32.7	50.5	0.379	0.632
**Burkholderia**	1.6	2.3	26.2	53.7	80.5	**0.016**	0.279
**Polaromonas**	23.2	12.5	24.8	21.1	71.0	0.897	0.970

**Novosphingobium**	32.6	25.5	23.7	23.3	50.5	0.379	0.632
**Spirosoma**	45.1	28.9	23.6	25.8	33.0	0.074	0.370
**Gp3 unclassified**	18.4	39.1	23.4	28.6	89.0	0.289	0.584
**Pseudomonas**	64.4	74.5	22.9	30.9	28.0	**0.041**	0.299
**Actinobacteria unclassified**	22.8	12.1	22.7	16.0	57.5	0.605	0.880
**Arenimonas**	33.9	23.4	22.6	22.1	35.5	0.098	0.392
**Sinobacteraceae unclassified**	5.6	5.9	21.9	28.6	88.0	0.118	0.411
**Planctomycetaceae unclassified**	16.0	28.2	20.6	19.6	91.0	0.245	0.584
**Brevundimonas**	15.8	12.6	20.2	23.0	72.5	0.836	0.970
**Proteobacteria unclassified**	18.6	13.4	20.1	17.9	67.5	1.000	1.000
**Gaiella**	11.9	12.3	18.3	29.2	63.0	0.887	0.970
**candidate division WPS.1 unclassified**	6.6	6.0	18.0	17.8	105.5	0.056	0.326
**Acidobacteria Gp4 unclassified**	26.2	27.4	17.9	15.5	41.0	0.170	0.505
**Blastocatella unclassified**	17.1	46.0	16.8	35.2	36.0	0.103	0.392
**Duganella**	22.7	7.5	16.7	15.7	93.0	0.065	0.345
**Terrimonas**	13.6	6.8	16.6	19.4	90.0	0.093	0.392
**Subdivision3 unclassified**	4.4	5.9	15.4	18.5	88.0	0.118	0.411
**Opitutus**	15.9	6.4	14.9	21.0	59.0	0.706	0.933
**Cytophagales unclassified**	16.1	12.5	14.6	20.2	46.0	0.266	0.584
**Roseomonas**	23.2	9.9	14.4	14.7	59.0	0.660	0.924
**Acidobacteria Gp3 unclassified**	8.1	17.3	14.1	20.5	79.0	0.293	0.584
**Dyadobacter**	14.2	12.0	13.7	18.6	56.0	0.842	0.970
**Cellvibrio**	36.9	31.5	13.6	12.7	43.2	0.023	0.279
**Acetobacteraceae unclassified**	14.3	18.7	13.6	20.7	68.0	0.670	0.924
**Gp4 unclassified**	1.1	1.9	13.3	40.4	53.5	0.270	0.584
**Rhizobium**	16.0	10.9	12.2	9.7	67.0	0.712	0.933
**Rhodopseudomonas**	17.3	7.7	12.1	10.4	64.5	0.877	0.970
**Nocardioidaceae unclassified**	14.4	11.0	12.1	10.2	53.5	0.733	0.946
**Flavisolibacter**	23.3	15.0	12.0	13.7	26.0	0.057	0.326
**Conexibacter**	24.6	40.5	11.1	15.1	49.0	0.338	0.615
**Chryseolinea unclassified**	7.0	6.6	11.1	12.3	54.0	0.576	0.869
**Roseiarcus**	0.4	1.1	10.4	23.7	43.2	0.128	0.425
**Pseudoxanthomonas**	10.2	11.5	10.0	8.6	67.0	0.979	1.000
**Cellulomonas**	9.9	9.4	8.6	6.9	66.0	0.938	0.988
**Phenylobacterium**	9.0	3.6	8.1	9.6	62.0	0.776	0.970
**Phycicoccus**	15.3	9.5	7.6	6.4	19.0	0.012	0.279
**Actinoplanes**	7.5	6.3	7.2	14.6	55.5	0.875	0.970
**Gp7 unclassified**	8.2	15.1	7.1	19.0	45.0	0.305	0.584
**Hyphomicrobium**	2.1	2.7	6.6	10.7	72.5	0.217	0.578
**Solirubrobacterales unclassified**	20.0	30.2	5.2	7.1	36.0	0.103	0.392
**Solirubrobacter**	5.7	4.0	5.1	7.9	60.5	0.637	0.910

C) Intervention vs standard, Day 28<br>Degrees of Freedom = 23	Mean standard	sd	Mean intervention	sd	Statistic	P value	P adjust*
Observed OTU richness	484.0	99.7	588.7	128.7	2.3	**0.029**	**0.04**
Chao richness index	533.0	134.9	670.9	156.9	2.4	**0.027**	**0.04**
Shannon total bacterial community	5.2	0.4	5.6	0.4	2.2	**0.040**	**0.04**
**CLASS LEVEL RICHNESS**							
Actinobacteria richness	72.5	16.2	75.8	16.4	0.5	0.626	0.939
Sphingobacteriia richness	67.4	17.8	74.4	17.2	1.0	0.333	0.777
Alphaproteobacteria richness	80.5	16.3	83.5	14.6	0.5	0.640	0.939
Betaproteobacteria richness	13.0	2.2	12.9	1.6	-0.1	0.939	0.939
Gammaproteobacteria richness	19.3	5.9	28.3	6.5	3.6	**0.002**	**0.024**
Flavobacteriia richness	13.0	2.2	12.9	1.6	-0.1	0.939	0.939
Cytophagia richness	39.7	15.5	28.6	16.7	-1.7	0.101	0.364
Acidobacteria Gp4 richness	13.0	2.2	12.9	1.6	-0.1	0.939	0.939
Deltaproteobacteria richness	9.8	5.1	14.1	8.0	1.7	0.104	0.364
Spartobacteria richness	13.0	2.2	12.9	1.6	-0.1	0.939	0.939
Bacilli richness	1.2	1.9	1.6	2.2	0.4	0.665	0.939
Gemmatimonadetes richness	13.0	2.2	12.9	1.6	-0.1	0.939	0.939
Acidobacteria Gp1 richness	3.4	3.5	11.3	20.3	1.5	0.150	0.419
Verrucomicrobiae richness	3.4	2.4	6	4.1	2.1	0.050	0.349
**CLASS LEVEL DIVERSITY**							
Actinobacteria Shannon	3.6	0.2	3.6	0.3	-0.2	0.850	0.850
Sphingobacteriia Shannon	3.3	0.3	3.6	0.3	2.7	**0.016**	0.074
Alphaproteobacteria Shannon	3.7	0.2	3.8	0.2	0.8	0.416	0.529
Betaproteobacteria Shannon	1.5	0.1	1.6	0.2	1.9	0.077	0.135
Gammaproteobacteria Shannon	2.3	0.3	2.6	0.3	2.6	**0.016**	0.074
Flavobacteriia Shannon	1.5	0.1	1.6	0.2	1.9	0.077	0.135
Cytophagia Shannon	3.1	0.4	2.6	0.6	-2.8	**0.010**	0.074
Acidobacteria Gp4 Shannon	1.5	0.1	1.6	0.2	1.9	0.077	0.135
Deltaproteobacteria Shannon	1.8	0.7	2.2	0.6	1.5	0.163	0.253
Spartobacteria Shannon	1.5	0.1	1.6	0.2	1.9	0.077	0.135
Bacilli Shannon	0.3	0.6	0.4	0.6	0.6	0.579	0.676
Gemmatimonadetes Shannon	1.5	0.1	1.6	0.2	1.9	0.077	0.135
Acidobacteria Gp1 Shannon	0.8	0.8	0.9	1.4	0.4	0.689	0.742
Verrucomicrobiae Shannon	0.9	0.6	1.2	0.7	1.0	0.316	0.442

*Benjamini-Hochberg correction

Table S4. Comparisons of bacterial richness and Shannon diversity on skin before the intervention period (Baseline A-C) and after the intervention period (Day-28 D-F) among three daycare groups (Intervention, standard and nature-oriented). Data is shown at total bacterial community, phylum and class levels (mean±standard deviation = sd). Analysis of covariance (ANCOVA) or Kruskal-Wallis test was used depending on Shapiro-Wilk test of normality. Tukey's HSD post hoc test was used when normality assumption was true and Dunn's test when normality assumption was violated. The upper part shows (**A**) **baseline** ANCOVA and Kruskal-Wallis tests and (**B**) post-hoc tests Tukey HSD and (**C**) Dunn's test. The lower part shows (**D**) **day-28** ANCOVA and Kruskal-Wallis tests and (**E**) post-hoc tests Tukey HSD and (**F**) Dunn's test. **Bold** font indicates significant differences ($p \leq 0.05$).

**A) Analysis of covariance**	**Intervention**		**Standard**		**Nature**		**ANCOVA[a]**		**Shapiro-Wilk**		**Kruskal-Wallis**	
**Baseline**	**mean**	**sd**	**mean**	**sd**	**mean**	**sd**	**F**	**P value**	**W**	**P value**	**X^2**	**P value**
**Total bacterial community**												
Observed OTU richness	52.8	29.3	56.6	31.7	65.9	39.6	1.1	0.347	1.0	0.099	1.3	0.531
Chao richness index	60.3	42.2	61.9	40.4	78.5	55.9	1.1	0.333	0.9	**0.005**	1.0	0.592
shannon	3.3	0.7	3.6	0.6	3.6	0.7	1.2	0.306	0.9	**0.001**	1.6	0.445
**DIVERSITY PHYLUM**												
Firmicutes Shannon	1.9	0.9	2.2	0.8	2.2	0.6	0.8	0.455	1.0	0.967	1.3	0.509
Proteobacteria Shannon	2.1	0.8	2.2	0.7	2.4	0.9	0.6	0.540	0.9	**0.007**	1.3	0.527
Actinobacteria Shannon	2.0	0.8	2.2	0.6	2.3	0.9	1.4	0.243	1.0	0.247	1.8	0.407
Bacteroidetes Shannon	1.2	0.9	1.2	0.9	1.4	1.0	0.3	0.736	0.9	**0.000**	0.7	0.722
**DIVERSITY CLASS**												
Bacilli Shannon	1.7	0.8	1.9	0.6	1.9	0.4	1.1	0.333	1.0	0.756	1.5	0.472
Gammaproteobacteria Shannon	1.5	0.7	1.4	0.7	1.3	0.6	0.6	0.572	1.0	**0.011**	1.2	0.547
Betaproteobacteria Shannon	1.0	0.8	1.2	0.7	1.5	0.7	2.2	0.117	1.0	0.053	3.4	0.179
Alphaproteobacteria Shannon	0.5	0.8	0.6	0.9	1.2	1.1	3.6	**0.033**	0.9	**0.000**	5.9	0.053
Clostridia Shannon	0.4	0.8	0.6	0.9	0.4	0.8	0.4	0.665	0.7	**0.000**	1.1	0.575
Bacteroidia Shannon	0.7	0.8	0.8	0.8	0.8	0.8	0.1	0.881	0.9	**0.000**	0.3	0.844
Fusobacteriia Shannon	0.4	0.6	0.5	0.7	0.4	0.7	0.1	0.899	0.7	**0.000**	0.4	0.832
Negativicutes Shannon	0.3	0.5	0.4	0.5	0.2	0.4	0.5	0.585	0.8	**0.000**	0.9	0.642
**RICHNESS PHYLUM**												
Firmicutes richness	16.3	15.3	16.0	10.4	14.9	8.8	0.1	0.936	0.9	**0.000**	0.3	0.852
Proteobacteria richness	14.2	10.7	13.4	8.2	20.1	14.6	2.2	0.120	0.9	**0.007**	2.4	0.295
Actinobacteria richness	12.1	8.8	14.4	12.0	18.3	14.6	2.0	0.147	0.9	**0.002**	1.3	0.512
Bacteroidetes richness	5.5	4.7	5.6	4.7	7.6	6.5	1.0	0.365	0.9	**0.001**	1.0	0.600
**CLASS**												
Bacilli richness	11.5	9.8	11.5	7.8	10.8	5.5	0.0	0.958	0.9	**0.000**	0.4	0.833
Gammaproteobacteria richness	6.9	4.8	5.6	3.5	6.0	3.6	0.5	0.599	1.0	0.115	0.2	0.897
Betaproteobacteria richness	4.5	3.7	4.5	2.4	7.1	5.2	3.1	**0.050**	0.9	**0.003**	3.8	0.149
Alphaproteobacteria richness	2.6	4.1	2.9	4.6	6.6	8.0	3.8	**0.027**	0.9	**0.000**	4.1	0.130
Clostridia richness	3.5	11.1	2.7	3.6	2.8	5.9	0.1	0.949	0.4	**0.000**	1.8	0.406

Bacteroidia richness	3.0	3.7	3.1	3.3	3.4	3.1	0.1	0.941	0.9	**0.000**	0.4	0.829
Fusobacteriia richness	1.6	2.0	2.2	3.1	1.6	2.0	0.4	0.661	0.8	**0.000**	0.0	0.989
Negativicutes richness	1.2	1.6	1.5	1.7	1.2	1.5	0.2	0.811	0.9	**0.000**	0.5	0.764

**B) POST-HOC TUKEY's HSD**	**Intervention vs Standard**		**Nature vs Standard**		**Nature vs Intervention**	
**Baseline**	**Mean difference**	**P value**	**Mean difference**	**P value**	**Mean difference**	**P value**
**Total bacterial community**						
Observed OTU richness	-3.843	0.922	9.302	0.688	13.144	0.329
Chao richness index	16.587	0.993	16.587	0.542	18.192	0.332
Shannon index	-0.546	0.561	0.056	0.973	0.284	0.350
**DIVERSITY PHYLUM**						
Firmicutes Shannon	-0.239	0.618	0.013	0.999	0.251	0.531
Proteobacteria Shannon	-0.087	0.935	0.162	0.832	0.249	0.522
Actinobacteria Shannon	-0.226	0.632	0.139	0.871	0.365	0.247
Bacteroidetes Shannon	-0.011	0.999	0.187	0.834	0.199	0.737
**DIVERSITY CLASS**						
Bacilli Shannon	-0.209	0.595	0.060	0.967	0.269	0.365
Gammaproteobacteria Shannon	0.148	0.752	-0.040	0.984	-0.188	0.582
Betaproteobacteria Shannon	-0.157	0.774	0.282	0.524	0.439	0.101
Alphaproteobacteria Shannon	-0.090	0.946	0.589	0.164	0.678	**0.030**
Clostridia Shannon	-0.235	0.654	-0.217	0.751	0.018	0.997
Bacteroidia Shannon	-0.074	0.951	0.036	0.991	0.110	0.877
Fusobacteriia Shannon	-0.094	0.889	-0.069	0.952	0.025	0.990
Negativicutes Shannon	-0.130	0.649	-0.164	0.584	-0.034	0.966
**RICHNESS PHYLUM**						
Firmicutes richness	0.314	0.997	-1.056	0.972	-1.370	0.930
Proteobacteria richness	0.771	0.972	6.683	0.200	5.911	0.151
Actinobacteria richness	-2.214	0.795	3.921	0.569	6.135	0.132
Bacteroidetes richness	-0.157	0.995	1.913	0.559	2.070	0.361
**RICHNESS CLASS**						
Bacilli richness	-0.543	1.000	-0.722	0.969	-0.679	0.959
Gammaproteobacteria richness	1.243	0.618	0.357	0.969	-0.886	0.747
Betaproteobacteria richness	-0.514	1.000	2.611	0.142	2.625	0.054
Alphaproteobacteria richness	-0.329	0.978	3.683	0.124	4.011	**0.027**
Clostridia richness	0.743	0.962	0.063	1.000	-0.679	0.962
Bacteroidia richness	-0.114	0.994	0.246	0.979	0.360	0.933
Fusobacteriia richness	-0.643	0.650	-0.603	0.742	0.040	0.998
Negativicutes richness	-0.829	0.791	-0.278	0.876	0.051	0.993

**C) POST-HOC DUNN'S TEST**	**Intervention vs Standard**		**Nature vs Standard**		**Nature vs Intervention**	
**Baseline**	**Dunn statistic**	**P value**	**Dunn statistic**	**P value**	**Dunn statistic**	**P value**
**Total bacterial community**						
Observed OTU richness	0.425	0.671	0.659	0.671	1.268	0.614
Chao richness index	0.374	0.708	0.629	0.708	1.177	0.708
Shannon index	0.748	0.649	0.455	0.649	1.371	0.511
**DIVERSITY PHYLUM**						
Firmicutes Shannon	1.154	0.405	0.125	0.900	1.104	0.405

Proteobacteria Shannon	0.257	0.797	0.586	0.797	0.996	0.797
Actinobacteria Shannon	0.271	0.786	0.801	0.635	1.274	0.608
Bacteroidetes Shannon	0.268	0.788	0.529	0.788	0.939	0.788
**DIVERSITY CLASS**						
Bacilli Shannon	0.672	0.752	0.212	0.832	0.992	0.752
Gammaproteobacteria Shannon	0.780	0.653	0.317	0.751	1.238	0.647
Betaproteobacteria Shannon	0.559	0.576	0.922	0.535	1.737	0.247
Alphaproteobacteria Shannon	0.413	0.679	1.533	0.188	2.324	0.060
Clostridia Shannon	1.256	0.627	0.806	0.630	0.383	0.702
Bacteroidia Shannon	0.554	0.869	0.108	0.914	0.735	0.869
Fusobacteriia Shannon	0.541	0.920	0.400	0.920	0.101	0.920
Negativicutes Shannon	0.804	0.632	0.829	0.632	0.138	0.890
**RICHNESS PHYLUM**						
Firmicutes richness	0.618	0.805	0.015	0.988	0.655	0.805
Proteobacteria richness	0.012	0.990	1.148	0.376	1.390	0.376
Actinobacteria richness	0.537	0.686	0.405	0.686	1.079	0.686
Bacteroidetes richness	0.346	0.730	0.639	0.730	1.158	0.730
Bacilli richness	0.234	0.886	0.144	0.886	0.430	0.886
**RICHNESS CLASS**						
Gammaproteobacteria richness	0.581	0.860	0.176	0.860	0.418	0.860
Betaproteobacteria richness	0.478	0.633	1.074	0.424	1.834	0.200
Alphaproteobacteria richness	0.537	0.591	1.100	0.407	1.930	0.161
Clostridia richness	0.963	0.503	0.364	0.716	1.495	0.405
Bacteroidia richness	0.499	0.844	0.197	0.844	0.784	0.844
Fusobacteriia richness	0.063	0.950	0.116	0.950	0.073	0.950
Negativicutes richness	0.723	0.776	0.285	0.776	0.439	0.776

**D) Analysis of covariance Day 28**	**Intervention**		**Standard**		**Nature**		**ANCOVA[a]**		**Shapiro-Wilk**		**Kruskal-Wallis**	
**Total bacterial community**	**Mean**	**sd**	**Mean**	**sd**	**Mean**	**sd**	**F**	**P value**	**W**	**P value**	**X^2**	**P value**
Observed OTU richness	62.3	39.7	40.8	14.9	69.1	37.8	2.6	0.087	0.9	**0.000**	4.6	0.102
Chao richness index	75.4	73.5	42.4	16.3	82.0	53.2	1.8	0.168	0.8	**0.000**	4.3	0.115
shannon	3.6	0.6	3.1	0.6	3.7	0.6	3.7	**0.030**	1.0	0.927	5.4	0.066
**DIVERSITY PHYLUM**												
Firmicutes Shannon	2.1	0.6	1.9	0.6	2.0	0.9	0.3	0.747	1.0	0.029	0.9	0.652
Proteobacteria Shannon	2.4	0.7	1.8	0.7	2.5	0.7	4.3	**0.018**	1.0	0.067	9.4	**0.009**
Actinobacteria Shannon	2.0	1.0	1.8	0.7	2.4	0.9	2.0	0.150	1.0	0.276	4.6	0.102
Bacteroidetes Shannon	1.3	0.9	1.0	0.7	1.5	0.8	1.8	0.181	1.0	0.139	2.7	0.260
**DIVERSITY CLASS**												
Bacilli Shannon	1.9	0.5	1.6	0.6	1.6	0.8	1.3	0.275	1.0	0.296	1.9	0.391
Gammaproteobacteria Shannon	1.6	0.7	1.1	0.7	1.4	0.7	2.7	0.073	0.9	**0.006**	6.4	**0.040**
Betaproteobacteria Shannon	1.3	0.8	0.9	0.6	1.5	0.7	2.7	0.077	1.0	**0.044**	8.7	**0.013**
Alphaproteobacteria Shannon	0.9	1.0	0.2	0.6	1.0	1.1	3.1	0.054	0.9	**0.002**	6.7	**0.036**
Clostridia Shannon	0.3	0.6	0.5	0.7	0.6	1.0	1.1	0.331	0.8	**0.000**	2.7	0.261
Bacteroidia Shannon	0.7	0.8	0.6	0.6	0.7	0.9	0.1	0.886	0.8	**0.000**	0.2	0.910
Fusobacteriia Shannon	0.6	0.7	0.4	0.5	0.5	0.6	0.4	0.677	0.9	**0.000**	0.8	0.680
Negativicutes Shannon	0.2	0.4	0.2	0.4	0.3	0.4	0.3	0.756	0.8	**0.000**	1.3	0.521
**RICHNESS PHYLUM**												

Firmicutes richness	14.8	9.9	14.2	8.3	15.9	12.2	0.1	0.899	0.9	**0.011**	0.0	0.989
Proteobacteria richness	19.9	15.4	9.1	4.6	19.8	13.0	3.4	**0.042**	0.9	**0.000**	9.4	**0.009**
Actinobacteria richness	15.6	16.7	9.3	4.8	19.4	13.1	2.1	0.136	0.8	**0.000**	3.9	0.142
Bacteroidetes richness	6.4	5.7	3.5	2.4	7.0	4.8	2.1	0.138	0.9	**0.000**	3.3	0.188
**RICHNESS CLASS**												
Bacilli richness	12.2	8.4	10.8	7.5	10.5	7.8	0.4	0.681	0.9	**0.000**	0.6	0.756
Gammaproteobacteria richness	7.8	5.3	4.5	2.8	6.9	5.1	1.8	0.168	0.9	**0.001**	4.4	0.111
Betaproteobacteria richness	6.6	5.9	3.3	2.2	7.0	4.5	2.5	0.090	0.9	**0.000**	8.0	**0.018**
Alphaproteobacteria richness	5.0	7.2	1.0	2.5	5.4	7.5	2.0	0.144	0.8	**0.000**	7.4	**0.025**
Clostridia richness	1.4	2.7	2.4	3.8	3.9	7.4	1.5	0.235	0.6	**0.000**	1.9	0.381
Bacteroidia richness	2.9	2.8	2.1	1.9	3.1	3.6	0.5	0.615	0.9	**0.000**	0.5	0.791
Fusobacteriia richness	2.5	2.5	1.4	1.3	2.0	2.8	0.9	0.409	0.9	**0.000**	1.8	0.401
Negativicutes richness	1.1	1.3	1.0	1.2	1.3	1.2	0.2	0.825	0.9	**0.000**	0.6	0.755

**E) POST-HOC TUKEY's HSD day 28**	**Intervention vs Standard**		**Nature vs Standard**		**Nature vs Intervention**	
**Total bacterial community**	**Mean difference**	**P value<br>P adjust***	**Mean difference**	**P value<br>P adjust***	**Mean difference**	**P value**
Observed OTU richness	21.541	0.168	28.283	0.075<br>0.113	6.742	0.794
Chao richness index	39.563	0.224	39.563	0.159<br>0.159	6.589	0.924
shannon	0.066	0.087	0.585	**0.024**<br>0.072	0.153	0.665
**DIVERSITY PHYLUM**						
Firmicutes Shannon	0.182	0.736<br>0.736	0.141	0.853<br>0.853	-0.041	0.980
Proteobacteria Shannon	0.645	**0.027**<br>0.108	0.720	**0.021**<br>0.084	0.075	0.934
Actinobacteria Shannon	0.228	0.724<br>0.736	0.599	0.157<br>0.209	0.371	0.342
Bacteroidetes Shannon	0.379	0.357<br>0.714	0.555	0.154<br>0.209	0.176	0.748
**DIVERSITY CLASS**						
Bacilli Shannon	0.282	0.410	0.024	0.994	-0.257	0.386
Gammaproteobacteria Shannon	0.551	0.053	0.369	0.309	-0.181	0.651
Betaproteobacteria Shannon	0.400	0.245	0.617	0.061	0.217	0.582
Alphaproteobacteria Shannon	0.731	0.064	0.792	0.063	0.060	0.975
Clostridia Shannon	-0.218	0.681	0.123	0.900	0.341	0.307
Bacteroidia Shannon	0.120	0.890	0.102	0.929	-0.017	0.997
Fusobacteriia Shannon	0.161	0.716	0.034	0.987	-0.127	0.766
Negativicutes Shannon	-0.032	0.970	0.069	0.890	0.101	0.689
**RICHNESS PHYLUM**						
Firmicutes richness	0.528	0.987<br>0.987	1.664	0.897	1.136	0.927
Proteobacteria richness	10.785	**0.038**<br>0.152	10.713	0.060	-0.073	1.000
Actinobacteria richness	6.244	0.363<br>0.484	10.061	0.110	3.817	0.613
Bacteroidetes richness	2.841	0.199<br>0.398	3.462	0.130	0.621	0.903
**RICHNESS CLASS**						
Bacilli richness	1.403	0.859	-0.296	0.994	-1.699	0.752
Gammaproteobacteria richness	3.220	0.117	2.409	0.348	-0.811	0.834
Betaproteobacteria richness	3.313	0.116	3.692	0.100	0.379	0.963
Alphaproteobacteria richness	4.034	0.156	4.421	0.147	0.387	0.978

Clostridia richness	-0.971	0.825	1.563	0.652	2.534	0.196
Bacteroidia richness	0.854	0.667	0.976	0.635	0.122	0.989
Fusobacteriia richness	1.098	0.378	0.615	0.766	-0.483	0.783
Negativicutes richness	0.069	0.985	0.263	0.832	0.194	0.861

**F) POST HOC DUNN'S TEST Day 28**	**Intervention vs Standard**			**Nature vs Standard**			**Nature vs Intervention**	
**Total bacterial community**	**Dunn statistic**	**P value**	**P adjust***	**Dunn statistic**	**P value**	**P adjust***	**Dunn statistic**	**P value**
Observed OTU richness	1.608	0.162		2.112	0.104		0.758	0.449
Chao richness index	1.505	0.199		2.064	0.117		0.816	0.415
shannon	1.664	0.144		2.321	0.061		0.949	0.342
**DIVERSITY PHYLUM**								
Firmicutes Shannon	0.815	0.415	0.415	0.857	0.392	0.392	0.123	0.902
Proteobacteria Shannon	2.579	**0.010**	**0.040**	2.920	**0.004**	**0.014**	0.644	0.520
Actinobacteria Shannon	0.914	0.361	0.415	2.069	**0.039**	0.077	1.489	0.136
Bacteroidetes Shannon	1.173	0.241	0.415	1.632	0.103	0.137	0.664	0.507
**DIVERSITY CLASS**								
Bacilli Shannon	1.236	0.216	0.413	0.337	0.736	0.954	0.987	0.324
Gammaproteobacteria Shannon	2.530	**0.005**	**0.041**	1.465	0.142	0.378	1.075	0.169
Betaproteobacteria Shannon	1.652	0.099	0.263	2.927	**0.003**	**0.027**	1.701	0.089
Alphaproteobacteria Shannon	2.379	**0.017**	0.069	2.293	0.022	0.087	0.106	0.915
Clostridia Shannon	1.130	0.258	0.413	0.187	0.852	0.954	1.507	0.132
Bacteroidia Shannon	0.433	0.665	0.665	0.246	0.806	0.954	0.190	0.849
Fusobacteriia Shannon	0.637	0.524	0.665	0.057	0.954	0.954	0.790	0.429
Negativicutes Shannon	0.439	0.660	0.665	0.528	0.598	0.954	1.141	0.254
**RICHNESS PHYLUM**								
Firmicutes richness	0.146	0.884	0.884	0.064	0.949	0.949	0.088	0.930
Proteobacteria richness	2.717	**0.007**	**0.026**	2.829	**0.005**	**0.019**	0.378	0.705
Actinobacteria richness	0.965	0.334	0.446	1.941	0.052	0.094	1.276	0.202
Bacteroidetes richness	1.359	0.174	0.349	1.808	0.071	0.094	0.669	0.504
**RICHNESS CLASS**								
Bacilli richness	0.412	0.680	0.777	0.216	0.829	0.864	0.729	0.466
Gammaproteobacteria richness	2.096	**0.036**	0.103	1.300	0.194	0.516	0.785	0.432
Betaproteobacteria richness	2.068	**0.039**	0.103	2.802	**0.005**	**0.041**	1.080	0.280
Alphaproteobacteria richness	2.607	**0.009**	0.073	2.262	0.024	0.095	0.189	0.850
Clostridia richness	0.486	0.627	0.777	0.688	0.491	0.849	1.390	0.165

[a] Degree of Freedom between groups = 2 and within groups = 58

*Benjamini-Hochberg correction

Table S5. Before-after comparisons **(**Wilcoxon-Signed Rank test) for bacterial diversity on skin at total bacterial community, and phylum and class levels among children in (**A**) intervention (n = 29), (**B**) standard (n = 14) and (**C**) nature-oriented daycare centers (n = 19) (Mean ± Standard Deviation = sd). **Bold** font indicates significant differences (p ≤ 0.05).

A) **Intervention**	**Mean before**	**sd before**	**Mean after**	**sd after**	**Statistic**	**P value**	**P adjust***
Observed OTU richness	53.3	29.1	63.5	41.0	239	0.110	
Chao richness index	59.9	40.1	77.5	75.8	257	0.105	
Shannon	3.3	0.8	3.6	0.6	268	0.059	
**DIVERSITY PHYLUM**							
Firmicutes Shannon	1.9	0.8	2.1	0.6	231	0.319	
Proteobacteria Shannon	2.2	0.8	2.4	0.7	233	0.296	
Actinobacteria Shannon	2.0	0.9	2.0	1.0	187	0.780	
Bacteroidetes Shannon	1.1	0.9	1.4	0.9	198	0.346	
Fusobacteria Shannon	0.5	0.6	0.6	0.7	102	0.486	
**DIVERSITY CLASS**							
Bacilli Shannon	1.8	0.8	1.9	0.5	225	0.394	0.879
Gammaproteobacteria Shannon	1.6	0.6	1.6	0.7	182	0.879	0.879
Betaproteobacteria Shannon	1.2	0.7	1.3	0.8	208	0.657	0.879
Alphaproteobacteria Shannon	0.6	0.9	1.0	1.0	127	**0.018**	0.144
Clostridia Shannon	0.3	0.4	0.3	0.6	42	0.844	0.879
Bacteroidia Shannon	0.6	0.7	0.7	0.8	97	0.632	0.879
Fusobacteriia Shannon	0.5	0.6	0.6	0.7	102	0.486	0.879
Negativicutes Shannon	0.2	0.4	0.2	0.4	37	0.756	0.879

**B) Standard**	**Mean before**	**sd before**	**Mean after**	**sd after**	**Statistic**	**P value**
Observed OTU richness	58.9	37.3	44.3	14.7	28	0.235
Chao richness index	69.2	52.8	47.8	19.1	28	0.235
Shannon	3.5	0.8	3.2	0.5	36	0.529
**DIVERSITY PHYLUM**						
Firmicutes Shannon	2.1	1.1	2.1	0.4	40	0.727
Proteobacteria Shannon	2.1	0.8	2.0	0.6	48	0.889
Actinobacteria Shannon	2.0	0.9	1.6	0.8	27	0.367
Bacteroidetes Shannon	1.2	0.9	1.1	0.7	30	0.505
Fusobacteria Shannon	0.5	0.7	0.6	0.6	25	0.813
**DIVERSITY CLASS**						
Bacilli Shannon	1.6	0.9	1.8	0.6	50	0.780
Gammaproteobacteria Shannon	1.2	0.6	1.3	0.6	57	0.442
Betaproteobacteria Shannon	1.2	0.7	1.2	0.5	48	0.889
Alphaproteobacteria Shannon	0.4	0.9	0.1	0.3	3	0.281
Clostridia Shannon	0.8	1.2	0.4	0.7	12	0.441
Bacteroidia Shannon	0.7	0.9	0.7	0.7	24	0.906

Fusobacteriia Shannon	0.5	0.7	0.6	0.6	25	0.813
Negativicutes Shannon	0.3	0.4	0.2	0.4	12	0.800
**C) Nature-oriented**	**Mean before**	**sd before**	**Mean after**	**sd after**	**Statistic**	**P value**
Observed OTU richness	69.5	41.1	73.7	39.2	64	0.864
Chao richness index	84.1	58.5	88.2	56.1	62	0.932
Shannon	3.7	0.8	3.8	0.6	69	0.629
**DIVERSITY PHYLUM**						
Firmicutes Shannon	2.2	0.6	2.2	0.9	57	0.887
Proteobacteria Shannon	2.4	1.0	2.6	0.8	68	0.670
Actinobacteria Shannon	2.3	0.9	2.6	0.8	74	0.443
Bacteroidetes Shannon	1.5	1.0	1.6	0.8	71	0.551
Fusobacteria Shannon	0.4	0.7	0.5	0.5	30	0.878
**DIVERSITY CLASS**						
Bacilli Shannon	1.9	0.4	1.7	0.8	49	0.551
Gammaproteobacteria Shannon	1.4	0.6	1.6	0.7	73	0.478
Betaproteobacteria Shannon	1.5	0.7	1.5	0.7	55	0.900
Alphaproteobacteria Shannon	1.3	1.1	1.1	1.1	34	0.724
Clostridia Shannon	0.5	0.8	0.7	1.1	34	0.541
Bacteroidia Shannon	0.8	0.7	0.8	0.9	29	0.756
Fusobacteriia Shannon	0.4	0.7	0.5	0.5	30	0.878
Negativicutes Shannon	0.3	0.5	0.3	0.4	28	0.594

Table S6. Gut bacterial before-after comparisons (Paired t-test) among A) intervention, B) nature-oriented and C) standard daycare children. Relative abundances are shown at order level and diversities at family level [Mean ± Standard Deviation (sd)].

**A) Intervention Degrees of Freedom = 22**	**Mean before**	**sd before**	**Mean after**	**sd after**	**Statistic**	**P value**	**P adjust***
**ORDER RELATIVE ABUNDANCE**							
Bacteroidales	1165.3	689.9	1490.9	503.2	2.2	0.039	0.256
Bifidobacteriales	181.1	98.1	190.2	167.8	0.2	0.820	0.888
Burkholderiales	17.5	26.7	17.0	15.1	-0.1	0.941	0.941
Clostridiales	2014.3	490.7	1717.7	401.1	-3.3	**0.003**	**0.043**
Coriobacteriales	45.1	35.3	63.1	66.2	1.6	0.127	0.383
Desulfovibrionales	1.3	3.5	2.8	4.7	1.2	0.236	0.383
Enterobacteriales	14.7	25.9	10.1	18.2	-0.9	0.404	0.478
Erysipelotrichales	11.3	12.5	23.0	29.0	1.8	0.083	0.358
Firmicutes_unclassified	41.1	75.2	22.8	36.2	-1.3	0.201	0.383
Lactobacillales	27.9	40.7	41.8	87.6	0.9	0.404	0.478
Pasteurellales	73.5	274.4	9.7	16.6	-1.1	0.277	0.401
Selenomonadales	94.1	99.5	143.8	166.5	1.3	0.225	0.383
Verrucomicrobiales	136.7	244.1	101.8	170.4	-1.3	0.219	0.383
**FAMILY DIVERSITIES**							

Bacteroidaceae Shannon	1.4	0.5	1.5	0.5	0.4	0.726	0.726
Bifidobacteriaceae Shannon	0.6	0.4	0.6	0.3	0.8	0.456	0.570
Lachnospiraceae Shannon	2.3	0.3	2.4	0.3	1.7	0.096	0.222
Prevotellaceae Shannon	0.2	0.4	0.1	0.3	-1.6	0.133	0.222
Ruminococcaceae Shannon	1.6	0.5	1.8	0.4	2.8	**0.010**	**0.050**
**B) Nature-oriented Degrees of Freedom = 19**	**Mean before**	**sd before**	**Mean after**	**sd after**	**Statistic**	**P value**	**P adjust***
**ORDER RELATIVE ABUNDANCE**							
Bacteroidales	1487.3	629.8	1764.3	840.9	1.6	0.133	
Bifidobacteriales	210.5	191.8	147.5	120.6	-2.1	0.051	
Burkholderiales	12.3	20.6	14.9	17.7	0.5	0.616	
Clostridiales	1910.3	515.2	1664.2	659.9	-1.5	0.147	
Coriobacteriales	42.7	37.8	37.6	39.0	-0.8	0.432	
Desulfovibrionales	1.5	3.9	1.7	3.9	0.2	0.819	
Enterobacteriales	4.4	9.3	15.7	47.2	1.0	0.312	
Erysipelotrichales	17.3	27.3	9.5	9.7	-1.7	0.109	
Firmicutes_unclassified	29.3	53.4	39.4	68.6	1.4	0.177	
Lactobacillales	29.1	44.7	18.9	36.4	-0.7	0.469	
Pasteurellales	17.2	32.8	8.6	12.8	-1.1	0.300	
Selenomonadales	42.8	73.5	65.1	109.0	1.0	0.347	
Verrucomicrobiales	31.9	60.9	39.4	79.8	0.5	0.602	
**FAMILY DIVERSITY**							
Bacteroidaceae Shannon	1.5	0.3	1.5	0.4	0.0	0.990	
Bifidobacteriaceae Shannon	0.6	0.3	0.5	0.4	-1.8	0.088	
Lachnospiraceae Shannon	2.5	0.4	2.5	0.3	1.2	0.244	
Prevotellaceae Shannon	0.2	0.4	0.3	0.4	1.2	0.254	
Ruminococcaceae Shannon	1.7	0.4	1.8	0.3	1.1	0.292	
**C) Standard Degrees of Freedom = 12**	**Mean before**	**sd before**	**Mean after**	**sd after**	**Statistic**	**P value**	**P adjust***
**ORDER RELATIVE ABUNDANCE**							
Bacteroidales	1060.2	791.1	1489.3	630.1	2.0	0.069	0.179
Bifidobacteriales	367.3	233.3	213.6	182.0	-2.1	0.059	0.179
Burkholderiales	1.4	2.6	13.6	11.8	3.4	**0.007**	0.091
Clostridiales	2123.5	642.3	1907.5	623.0	-1.2	0.260	0.483
Coriobacteriales	77.3	71.5	42.5	46.5	-2.6	**0.029**	0.179
Desulfovibrionales	1.3	4.2	2.1	3.3	0.7	0.502	0.670
Enterobacteriales	11.3	15.2	6.9	20.0	-0.5	0.617	0.670
Erysipelotrichales	24.5	25.9	12.6	12.3	-1.7	0.113	0.245
Firmicutes_unclassified	35.6	34.2	44.7	78.4	0.5	0.618	0.670
Lactobacillales	31.4	48.3	4.6	8.3	-2.1	0.060	0.179
Pasteurellales	2.5	5.2	3.1	4.4	0.4	0.718	0.718
Selenomonadales	39.0	65.1	55.1	115.5	0.6	0.580	0.670
Verrucomicrobiales	53.6	120.9	30.4	74.1	-0.5	0.614	0.670
**FAMILY DIVERSITY**							
Bacteroidaceae Shannon	1.3	0.3	1.5	0.4	2.3	**0.044**	0.219
Bifidobacteriaceae Shannon	0.5	0.3	0.5	0.3	0.1	0.902	0.902
Lachnospiraceae Shannon	2.5	0.2	2.4	0.4	-0.5	0.634	0.793

Prevotellaceae Shannon	0.0	0.1	0.1	0.2	1.3	0.227	0.567
Ruminococcaceae Shannon	1.9	0.4	1.8	0.3	-0.6	0.560	0.793

*Benjamini-Hochberg correction

Table S7. Correlations between diversity changes on skin microbiota and plasma cytokine concentrations.

(**A**) Increase in skin Gammaproteobacterial Alpha diversity during the 28-day intervention period was associated with arise in plasma TGF-β1 concentration when all children (n = 26) were analyzed with linear mixed model. Among the intervention daycare center children (n = 12), (**B**) this diversity increase was also associated with a decrease in plasma IL-17A levels and (**C**) increase in total Treg cell levels. (**D**) Among the nature-oriented daycare center children (n = 10), the high Gammaproteobacterial diversity was associated with an increase in IL-10 expression, while Proteobacterial diversity was associated with a decrease in IL-10 expression. (**E**) Among the standard daycare center children (n = 6), the Gammaproteobacterial diversity change was mostly negative, and it was associated with TGF-β1 plasma concentration. Thus, the change in TGF-β1 plasma concentration was mostly negative. (**F**) Linear mixed-effect model showed that among children in nature-oriented daycare centers (n = 14), the higher abundance of Faecalibacterium prausnitzii (Otu000007) was associated with lower plasma levels of IL-17A and Otu000008 (unknown Faecalibacterium) with higher plasma levels of IL-17A. (**G**) The latter association was observed also within standard daycare children (n = 8). (**H**) Linear mixed-effect model showed that among nature-oriented daycare center children (n = 10), the decrease in IL-17A during the trial was associated with the decrease in the relative abundance of *Dorea* and *Romboutsia*, and increase in *Anaerostipes*.

**A) All children: TGF-β1**	**Estimate**	**Std. Error**	**t value**	**P value**	**P adjusted***
**Gammaproteobacteria**	2.601	0.904	2.877	**0.004**	**0.012**
**B) Intervention children: IL-17A**	**Estimate**	**Std. Error**	**t value**	**P value**	**P adjusted***
**Gammaproteobacteria**	-0.885	0.283	-3.132	**0.002**	**0.003**
**C) Treg cells**	**Estimate**	**Std. Error**	**t value**	**P value**	
**Intervention: Gammaproteobacteria**	1.238	0.513	2.413	**0.016**	
**Standard: Gammaproteobacteria**	-0.281	0.300	-0.936	0.349	

D) Nature-oriented children: IL-10	Estimate	Std. Error	t value	P value	P adjusted*
**Gammaproteobacteria**	3.539	0.715	4.947	**7.5 x 10^{-7}**	**2.3 x 10^{-6}**
**Proteobacteria**	-2.278	0.644	-3.536	**0.0004**	**0.0006**

E) Standard children: TGF-β1	Estimate	Std. Error	t value	P value	P adjusted*
**Gammaproteobacteria**	4.388	1.598	2.745	**0.006**	**0.012**

F) Nature-oriented children	Estimate	Std. Error	t value	P value	P adjusted*
**Otu000007**	-0.023	0.006	-3.885	**0.000**	**0.004**
**Otu000008**	0.013	0.004	3.470	**0.001**	**0.009**

G) Standard children	Estimate	Std. Error	t value	P value	P adjusted*
**Otu000008**	0.033	0.012	3.067	**0.002**	**0.038**

H) Nature-oriented children: IL-17A	Estimate	Std. Error	t value	P value	P adjusted*
**Dorea**	0.033	0.011	2.953	**0.003**	**0.027**
**Romboutsia**	0.0381	0.008	4.794	**1.6 x 10^{-6}**	**5.7 x 10^{-5}**
**Anaerostipes**	-0.013	0.004	-3.324	**0.0009**	**0.010**

*Benjamini-Hochberg correction

Table S8. Correlation between stool *Faecalibacterium* community composition and plasma cytokine levels.

Community composition of *Faecalibacterium* was associated with the IL-17A concentration in the plasma (n = 55) after but not before the 28-day intervention. No other associations between the measured plasma cytokines and *Faecalibacterium* community composition were found. Non-metric multidimensional scaling (NMDS) with Bray-Curtis metric was used to score the OTUs onto an ordination (NMDS coordinates 1 and 2) and correlation with corresponding cytokine expression levels was assessed using function *envfit* in *vegan* package in R computing environment.

**Before**	**NMDS 1**	**NMDS 2**	**r^2**	**P-value**	
**IL-10**	0.292	0.956	0.011	0.794	
**IL-17A**	-0.517	0.856	0.076	0.179	
**TGF-β1**	-0.302	0.953	0.045	0.362	
**After**	**NMDS 1**	**NMDS 2**	**r^2**	**P-value**	**P adjust***
**IL-10**	-0.950	-0.311	0.048	0.354	0.531
**IL-17A**	-0.884	-0.468	0.214	**0.015**	**0.045**
**TGF-β1**	0.918	0.397	0.025	0.585	0.585

*Benjamini-Hochberg correction

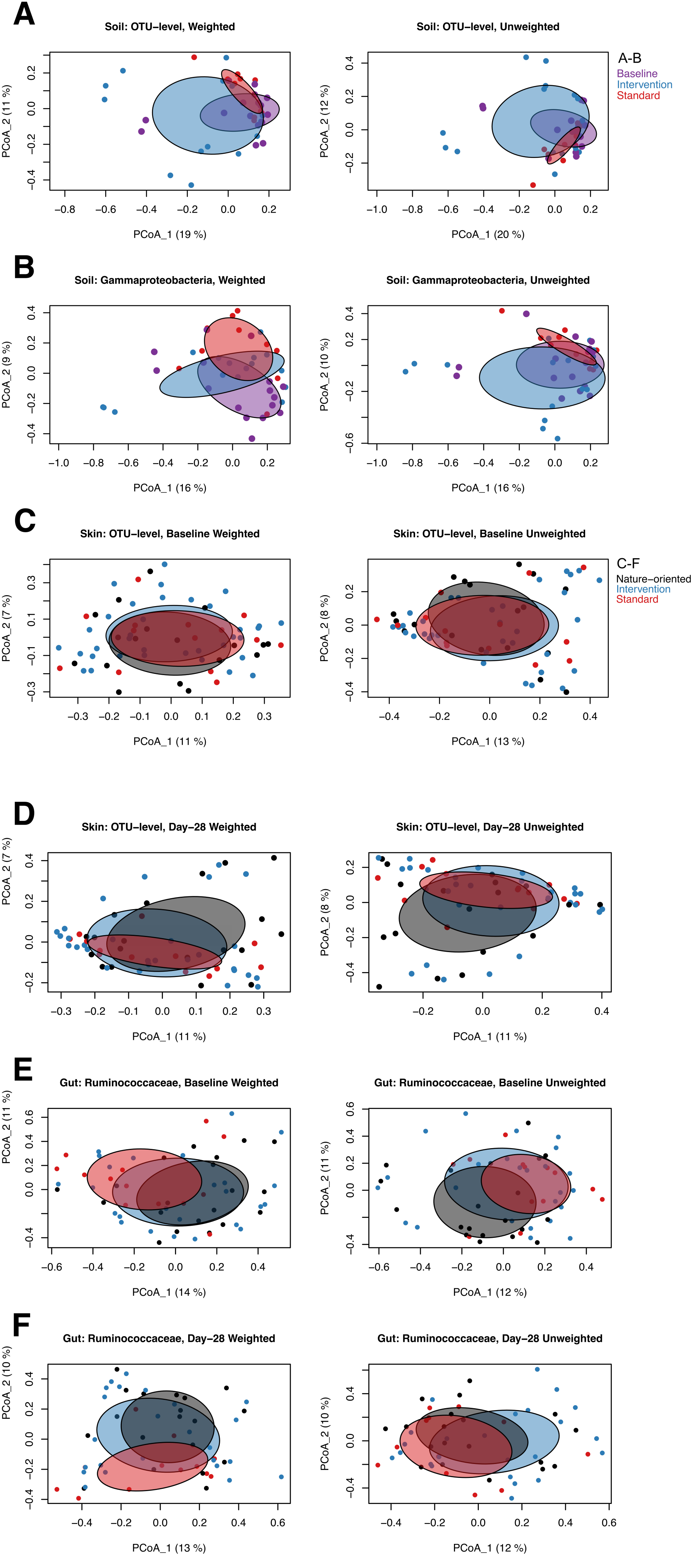
A
Soil: OTU−level, Weighted
PCoA_1 (19 %)
PCoA_2 (11 %)
Soil: OTU−level, Unweighted
PCoA_1 (20 %)
PCoA_2 (12 %)
A-B
Baseline
Intervention
Standard
B
Soil: Gammaproteobacteria, Weighted
PCoA_1 (16 %)
PCoA_2 (9 %)
Soil: Gammaproteobacteria, Unweighted
PCoA_1 (16 %)
PCoA_2 (10 %)
C
Skin: OTU−level, Baseline Weighted
PCoA_1 (11 %)
PCoA_2 (7 %)
Skin: OTU−level, Baseline Unweighted
PCoA_1 (13 %)
PCoA_2 (8 %)
C-F
Nature−oriented
Intervention
Standard
D
Skin: OTU−level, Day−28 Weighted
PCoA_1 (11 %)
PCoA_2 (7 %)
Skin: OTU−level, Day−28 Unweighted
PCoA_1 (11 %)
PCoA_2 (8 %)
E
Gut: Ruminococcaceae, Baseline Weighted
PCoA_1 (14 %)
PCoA_2 (11 %)
Gut: Ruminococcaceae, Baseline Unweighted
PCoA_1 (12 %)
PCoA_2 (11 %)
F
Gut: Ruminococcaceae, Day−28 Weighted
PCoA_1 (13 %)
PCoA_2 (10 %)
Gut: Ruminococcaceae, Day−28 Unweighted
PCoA_1 (12 %)
PCoA_2 (10 %)

Fig. S1. Principal Coordinate Analysis (PCoA) for differences and shifts in bacterial communities A-B) in soil, B-C) on skin and E-F) in the gut. PCoA plots were calculated with dissimilarity indices with Bray-Curtis metric for weighted (abundance data) and unweighted (presence/absence data) data sets. For details of significant differences, please see Table S2. After the intervention period, **(A)** standard and intervention yards had different weighted and unweighted total bacterial and **(B)** Gammaproteobacterial community composition in soil. **(C)** Skin total bacterial community differed before intervention period between children in nature-oriented and standard daycares, **(D)** but not after intervention. In the gut, the sole difference at baseline was found **(E)** between nature-oriented and standard children in Ruminococcaceae community before intervention, while **(F)** after the intervention period Ruminococcaceae community was different additionally between the intervention and standard children.

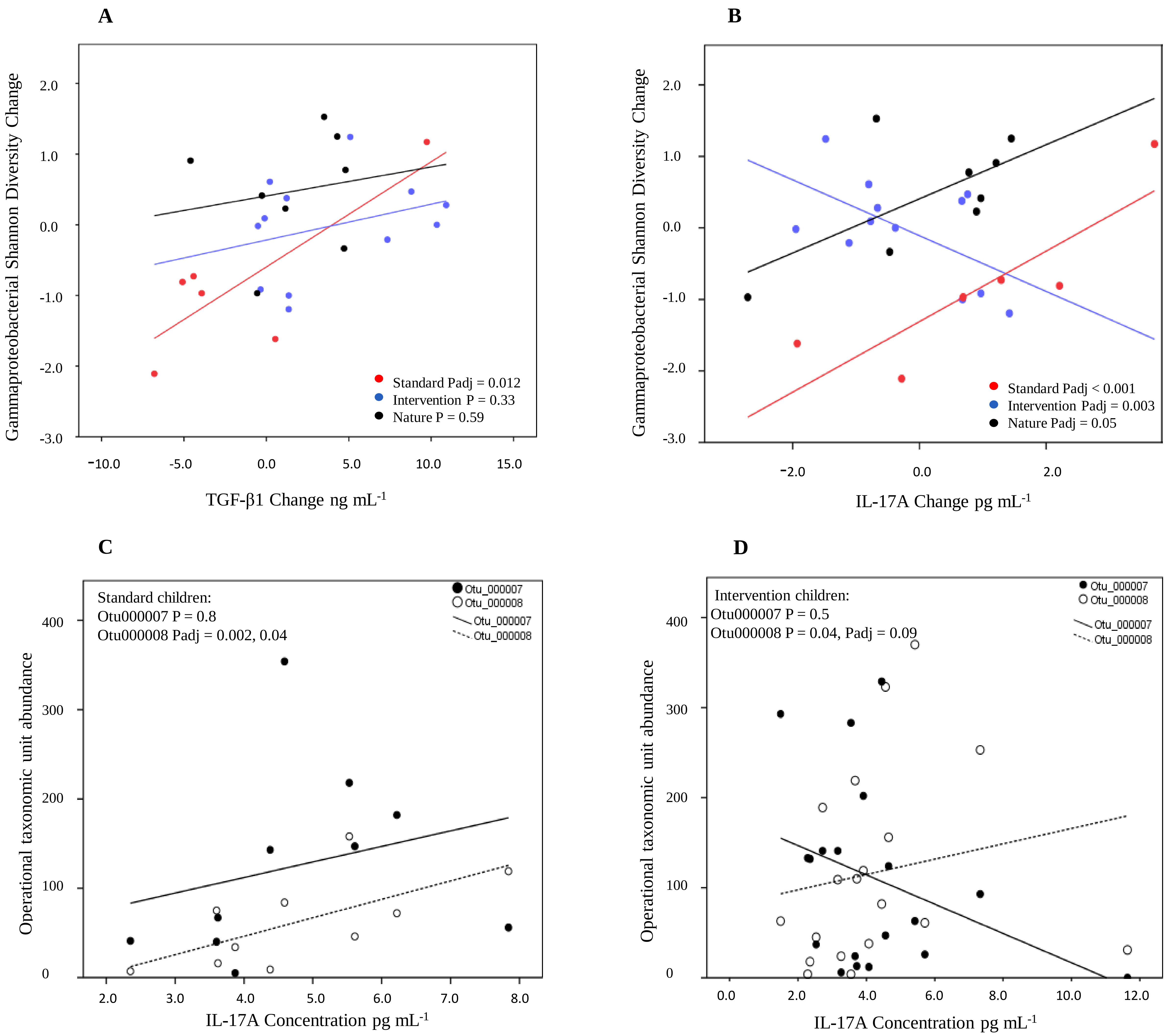
A
Gammaproteobacterial Shannon Diversity Change
2.0
1.0
0.0
-1.0
-2.0
-3.0
-10.0
-5.0
0.0
5.0
10.0
15.0
TGF-β1 Change ng mL-1
Standard Padj = 0.012
Intervention P = 0.33
Nature P = 0.59
B
Gammaproteobacterial Shannon Diversity Change
-2.0
0.0
2.0
IL-17A Change pg mL-1
Standard Padj < 0.001
Intervention Padj = 0.003
Nature Padj = 0.05
C
Standard children:
Otu000007 P = 0.8
Otu000008 Padj = 0.002, 0.04
Otu_000007
Otu_000008
Operational taxonomic unit abundance
400
300
200
100
0
2.0
3.0
4.0
5.0
6.0
7.0
8.0
IL-17A Concentration pg mL-1
D
Intervention children:
Otu000007 P = 0.5
Otu000008 P = 0.04, Padj = 0.09
Otu_000007
Otu_000008
Operational taxonomic unit abundance
0.0
2.0
4.0
6.0
8.0
10.0
12.0
IL-17A Concentration pg mL-1

Fig. S2. Associations between change in A-B) skin Gammaproteobacterial diversity and C-D) gut Faecalibacterium OTUs, and immune markers. Skin Gammaproteobacterial diversity change was associated with plasma **(A)** TGF-β1 (ng mL^{-1}) and **(B)** IL-17A (pg mL^{-1}) change among children in standard, intervention and nature-oriented daycares. **(C)** Among standard and **(D)** intervention children, *Faecalibacterium* Otu000008 was associated with an increase in IL-17A concentration (results from end of trial). Skin alpha diversity (Shannon index) and immune markers were measured on days 0 and 28 (change = day 28 – day 0).
